# Utilization of mechanical power and associations with clinical outcomes in brain injured patients: a secondary analysis of the extubation strategies in neuro-intensive care unit patients and associations with outcome (ENIO) trial

**DOI:** 10.1186/s13054-023-04410-z

**Published:** 2023-04-20

**Authors:** Sarah Wahlster, Monisha Sharma, Shaurya Taran, James A. Town, Robert D. Stevens, Raphaël Cinotti, Karim Asehoune, Paolo Pelosi, Chiara Robba, ENIO Study Group Collaborators, Paër-sélim Abback, Anaïs Codorniu, Giuseppe Citerio, Vittoria Ludovica Sala, Marinella Astuto, Eleonora Tringali, Daniela Alampi, Monica Rocco, Jessica Giuseppina Maugeri, Agrippino Bellissima, Matteo Filippini, Nicoletta Lazzeri, Andrea Cortegiani, Mariachiara Ippolito, Denise Battaglini, Patrick Biston, Mohamed Fathi Al-Gharyani, Russell Chabanne, Léo Astier, Benjamin Soyer, Samuel Gaugain, Alice Zimmerli, Urs Pietsch, Miodrag Filipovic, Giovanna Brandi, Giulio Bicciato, Ainhoa Serrano, Berta Monleon, Peter van Vliet, Benjamin Marcel Gerretsen, Iris Xochitl Ortiz-Macias, Jun Oto, Noriya Enomoto, Tomomichi Matsuda, Nobutaka Masui, Pierre Garçon, Jonathan Zarka, Wytze J. Vermeijden, Alexander Daniel Cornet, Sergio Reyes Inurrigarro, Rafael Cirino Lara Domínguez, Maria Mercedes Bellini, Maria Milagros Gomez Haedo, Laura Lamot, Jose Orquera, Matthieu Biais, Delphine Georges, Arvind Baronia, Roberto Carlos Miranda-Ackerman, Francisco José Barbosa-Camacho, John Porter, Miguel Lopez-Morales, Thomas Geeraerts, Baptiste Compagnon, David Pérez-Torres, Estefanía Prol-Silva, Hana Basheer Yahya, Ala Khaled, Mohamed Ghula, Cracchiolo Neville Andrea, Palma Maria Daniela, Cristian Deana, Luigi Vetrugno, Manuel J. Rivera Chavez, Rocio Mendoza Trujillo, Vincent Legros, Benjamin Brochet, Olivier Huet, Marie Geslain, Mathieu van der Jagt, Job van Steenkiste, Hazem Ahmed, Alexander Edward Coombs, Jessie Welbourne, Ana Alicia Velarde Pineda, Víctor Hugo Nubert Castillo, Mohammed A. Azab, Ahmed Y. Azzam, David Michael Paul van Meenen, Gilberto Adrian Gasca, Alfredo Arellano, Forttino Galicia-Espinosa, José Carlos García-Ramos, Ghanshyam Yadav, Amarendra Kumar Jha, Vincent Robert-Edan, Pierre-Andre Rodie-Talbere, Gaurav Jain, Sagarika Panda, Sonika Agarwal, Yashbir Deewan, Gilberto Adrian Gasca, Alfredo Arellano, Syed Tariq Reza, Md.Mozaffer Hossain, Christos Papadas, Vasiliki Chantziara, Chrysanthi Sklavou, Yannick Hourmant, Nicolas Grillot, Job van Steenkiste, Mathieu van der Jagt, Romain Pirracchio, Abdelraouf Akkari, Mohamed Abdelaty, Ahmed Hashim, Yoann Launey, Elodie Masseret, Sigismond Lasocki, Soizic Gergaud, Nicolas Mouclier, Sulekha Saxena, Avinash Agrawal, Shakti Bedanta Mishra, Samir Samal, Julio Cesar Mijangos, Mattias Haënggi, Mohan Gurjar, Marcus J. Schultz, Callum Kaye, Daniela Agustin Godoy, Pablo Alvarez, Aikaterini Ioakeimidou, Yoshitoyo Ueno, Rafael Badenes, Abdurrahmaan Ali Suei Elbuzidi, Michaël Piagnerelli, Muhammed Elhadi, Syed Tariq Reza, Jean Catherine Digitale, Nicholas Fong, Ricardo Campos Cerda, Norma de la Torre Peredo

**Affiliations:** 1 Neurocritical Care, Department of Neurology, Harborview Medical Center, University of Washington, Box 359702, 325 9th Avenue, WA 98104-2499 Seattle, USA; 2 Department of Neurological Surgery, Harborview Medical Center, University of Washington, Seattle, USA; 3 Department of Anesthesiology and Pain Medicine, Harborview Medical Center, University of Washington, Seattle, USA; 4 Department of Global Health, University of Washington, Seattle, USA; 5 Department of Neurology, Massachusetts General Hospital, Harvard Medical School, Boston, MA USA; 6 Interdepartmental Division of Critical Care Medicine, University of Toronto, Toronto, ON Canada; 7 Division of Pulmonary, Critical Care and Sleep Medicine, Department of Medicine, University of Washington, Seattle, USA; 8 Department of Anesthesiology and Critical Care Medicine, Johns Hopkins University School of Medicine, Baltimore, MD USA; 9 Department of Anesthesiology and Critical Care, CHU Nantes, Nantes Université, Nantes, France; 10 Department of Surgical Sciences and Integrated Diagnostics, University of Genoa, Genoa, Italy; 11 Anesthesia and Critical Care, San Martino Policlinico Hospital, IRCCS for Oncology and Neurosciences, Genoa, Italy; 12 Department of Anesthesiology and Critical Care, Beaujon Hospital, DMU Parabol, AP-HP.Nord, 100 Boulevard du General Leclerc, Clichy, 92100 Paris, France; 13 Neurointensive Care Unit, Ospedale San Gerardo, Azienda Socio-Sanitaria Territoriale di Monza, 23 Via Aliprandi, 20900 Monza, Italy; 14 NeuroIntensive Care Unit, ASST-Monza, Via Pergolesi, Monza, Italy; 15 Anesthesia and Intensive Care Unit, A.O.U. Policlinico “G. Rodolico - S. Marco”, Via Santa Sofia 78, 95123 Catania, Italy; 16 Sapienza Rome University, A.O.U. Sant’Andrea, 1035/1039, Via di Grottarossa, 00189 Rome, Italy; 17 ARNAS Garibaldi Catania, Piazza S.Maria di Gesu’ 5, 95123 Catania, Italy; 18 University Division of Anesthesiology and Critical Care Medicine, ASST Spedali Civili, Piazzale Spedali Civili, 1 - Brescia, 25123 Brescia, Italy; 19 Policlinico Paolo Giaccone, Università Degli Studi Di Palermo, Via del Vespro 129, 90127 Palermo, Italy; 20 San Martino Policlinico Hospital, IRCCS for Oncology and Neurosciences, 10 Largo Rosanna Benzi, 16100 Genoa, Italy; 21 CHU Charleroi - Hôpital Civil Marie-Curie, 140 Chaussée de Bruxelles, 6042 Lodelinsart, Charleroi, Belgium; 22 Benghazi Medical Center, Eastern Selmain, Benghazi, NA Libya; 23 Perioperative Medicine Department, Clermont-Ferrand University Hospital, Neurocritical Care Unit, 58 rue Montalembert, 63000 Clermont-Ferrand, France; 24 Department of Anesthesia and Critical Care, AP-HP, Hôpital Lariboisière, DMU Parabol, 2 rue Ambroise Paré, 75010 Paris, France; 25 Department of Intensive Care Medicine, Inselspital, Bern University Hospital, University of Bern, 3010 Freiburgstrasse, Bern, Switzerland; 26 Department of Anaesthesiology and Intensive Care Medicine, Cantonal Hospital St Gallen, Rorschacher Strasse 95, 9007 St. Gallen, Switzerland; 27 Institute for Intensive Care Medicine, University Hospital of Zurich, Rämistrasse 100, 8091 Zurich, Switzerland; 28 Hospital Clinico Universitario Valencia, Avenida Blasco Ibañez, 17, 46010 Valencia, Spain; 29 Haaglanden Medical Center, Lijnbaan 32, 2512 VA The Hague, The Netherlands; 30 Hospital Civil de Guadalajara “Fray Antonio Alcalde”, Hospital No. 278, Col. El Retiro, 44280 Guadalajara, Mexico; 31 Tokushima University Hospital, 2-50-1, Kuramoto-cho, Tokushima-shi, Tokushima, 7700042 Japan; 32 Tokushima Prefectural Central Hospital, 1-10-3, Kuramoto-cho, Tokushima-shi, Tokushima, 7708539 Japan; 33 Sapporo Higashi Tokushukai Hospital, 3-1, Kita 33-jo Higashi 14-chome, Higashi-ku, Sapporo, 0650033 Japan; 34 Service de Réanimation, 2-4 Cours de la Gondoire, 77600 Jossigny, France; 35 Department of Intensive Care, Medisch Spectrum Twente MST, Koningsplein 1, 7512 KZ Enschede, The Netherlands; 36 UMAE Hospital de Traumatologia y Ortopedia IMSS, Diagonal Defensores de la Republica esquin 6 poniente, 72090 Puebla, Mexico; 37 Hospital Maciel, 25 de Mayo 174, 11000 Montevideo, Uruguay; 38 Hospital Municipal Leonidas Lucero, Bahia Blanca, B8000 Buenos Aires, Argentina; 39 Sanatorio Pasteur, Chacabuco 675, 4700 Catamarca, Argentina; 40 Pellegrin SAR Tripode, Place Amelie Raba Leon, 33076 Bordeaux, France; 41 Department of Critical Care Medicine, Sanjay Gandhi Postgraduate Institute of Medical Sciences (SGPGIMS), Lucknow, 226014 India; 42 Hospital San Javier, Av Pablo Casals 640, Prados Providencia, 44670 Guadalajara, Jalisco Mexico; 43 St George’s Hospital, Blackshaw Road, London, SW17 0QT UK; 44 Toulouse University Hospital, Place du Dr Baylac, 31059 Toulouse, France; 45 Servicio de Medicina Intensiva, Hospital Universitario Río Hortega, Calle Dulzaina, 2, 47012 Valladolid, Spain; 46 Zliten Medical Centre, Khaam, 218 Zliten, Libya; 47 Abo Selim Trauma Hospital, Abo Selim Main, Tripoli, Libya; 48 Terapia Intensiva con Trauma Center, ARNAS Ospedale Civico Palermo, Piazza n Leotta 4, 90127 Palermo, Italy; 49 Academic Hospital of Udine, Piazzale S.Maria della Misericordia, 15, 33100 UdineChieti, Italy; 50 University of Chieti-Pescara, Via dei Vestini n 33, Chieti, Italy; 51 Hospital de Alta Especialidad del Bajio, Blvd. Milenio #130 Col. San Carlos la Roncha, 37660 Leon, Guanajuato, Mexico; 52 Department of Anesthesiology and Critical Care, University Hospital of Reims, Hopital Maison Blanche, 45 Rue Cognacq Jay, 51100 Reims, France; 53 Department of Anesthesiology and Critical Care, La Cavale Blanche, Boulevard Tanguy Prigent, 29200 Brest, France; 54 Erasmus MC, Dr Molewaterplein 40, 3015CE Rotterdam, The Netherlands; 55 Seoul Clinic, 20th Ramadan Road, Tripoli, Libya; 56 Department of Intensive Care Medicine, University Hospital Plymouth, Derriford Road, Plymouth, PL6 8DH Devon UK; 57 Hospital General Regional # 180 IMSS, Carretera a San Sebastian # 1000 Col. Las cumbres 2 Tlajomulco de Zúñiga, 45650 Guadalajara, Mexico; 58 Cairo University, Cairo, Giza, 12613 Egypt; 59 Amsterdam UMC, Meibergdreef 9, 1105AZ Amsterdam, The Netherlands; 60 Hospital Regional de Alta Especialidad de Ixtapaluca, Carretera Federal Mexico, Puebla Km. 34.5, Pueblo de Zoquiapan, 56530 Ixtapaluca, Mexico; 61 UMAE Hospital de Traumatología y Ortopedia No 21, IMSS Monterrey, Av. J.M. Pino Suárez S/N Esq. 15 de Mayo, 64000 Monterrey, Mexico; 62 Department of Anesthesia, Trauma ICU, IMS, BHU, Varanasi, 221005 India; 63 Nantes-Laennec, 1 Bd Jacques Monod, 44093 Nantes, France; 64 Critical Care Unit, Dept. of Anaesthesiology and Critical Care, All India Institute of Medical Sciences Rishikesh, Virbhadra Marg, Rishikesh, 249203 India; 65 HIMS, B18/8, HIMS Campus, Dehradun, 248140 India; 66 Department of Anaesthesia, Analgesia, Palliative and Intensive Care, Dhaka Medical College Hospital, Dhaka, 1000 Bangladesh; 67 ICU of Asklepieio G.H.A, V.Paulou 1, 16673 Athens, Greece; 68 Saint Savvas Hospital, 151 Alexandras Avenue, 11522 Athens, Greece; 69 Department of Anesthesiology and Critical Care, Hôtel-Dieu, 1 Place Alexis Ricordeau, 44093 Nantes, France; 70 Erasmus Medical Centre, Doctor Molewaterplein 40, 3015GD Rotterdam, The Netherlands; 71 Department of Anesthesia and Perioperative Care, University of California, UCSF, 1001 Potrero Ave, San Francisco, CA 94110 USA; 72 Qatar-1, HMC-Doha-Qatar, Doha, NA Qatar; 73 Qatar-2, HMC-Doha-Qatar, Doha, NA Qatar; 74 Department of Anesthesiology and Critical Care, Hopital Pontchaillou, 2 Rue Henri Le Guilloux, 35000 Rennes, France; 75 Department of Anesthesiology and Critical Care, University of Angers, 4 Rue Larrey, 49100 Angers, France; 76 Department of Critical Care Medicine, King George’s Medical University, Lucknow, 226014 India; 77 IMS and SUM Hospital, K8 Kalinga Nagar, Bhubaneswar, 751003 India; 78 Inselspital, Bern University Hospital, 3010 Freiburgstrasse, Bern, Switzerland; 79 Department of Intensive Care, Amsterdam University Medical Centers, 1105 AZ Amsterdam, The Netherlands; 80 Aberdeen Royal Infirmary, Foresthill, Aberdeen, AB25 2ZN UK; 81 Hospital Maciel, ASSE, Street 25 de Mayo 174, 11000 Montevideo, Uruguay; 82 Asklepieio G.H.A, V.Paulou 1, 16673 Athens, Greece; 83 Hospital Clínico Universitario Valencia, Valencia, Spain; 84 Qatar-1, Hamad Medical Corporation, Doha, Qatar; 85 Hôpital Civil Marie-Curie, Université Libre de Bruxelles, 140 Chaussée de Bruxelles, 6042 Lodelinsart, Charleroi, Belgium; 86 Faculty of Medicine, University of Tripoli, University Road, Furnaj, 13275 Tripoli, Libya; 87 Dhaka Medical College Hospital, Dhaka, 1000 Bangladesh; 88 University of California, UCSF, 550 16Th Street, San Francisco, CA 94158 USA; 89 University of California, UCSF, 1001 Potrero Ave, San Francisco, CA 94110 USA; 90 Critical Care Unit, Hospital General Regional No. 46, Instituto Mexicano del Seguro Social, 2063 Lazaro Cárdenas Av, 44910 Guadalajara, Mexico

**Keywords:** Acute brain injury, Mechanical power, Acute respiratory distress syndrome, Mechanical ventilation, Traumatic brain injury, Subarachnoid hemorrhage, Acute ischemic stroke, Intracranial hemorrhage

## Abstract

**Background:**

There is insufficient evidence to guide ventilatory targets in acute brain injury (ABI). Recent studies have shown associations between mechanical power (MP) and mortality in critical care populations. We aimed to describe MP in ventilated patients with ABI, and evaluate associations between MP and clinical outcomes.

**Methods:**

In this preplanned, secondary analysis of a prospective, multi-center, observational cohort study (ENIO, NCT03400904), we included adult patients with ABI (Glasgow Coma Scale ≤ 12 before intubation) who required mechanical ventilation (MV) ≥ 24 h. Using multivariable log binomial regressions, we separately assessed associations between MP on hospital day (HD)1, HD3, HD7 and clinical outcomes: hospital mortality, need for reintubation, tracheostomy placement, and development of acute respiratory distress syndrome (ARDS).

**Results:**

We included 1217 patients (mean age 51.2 years [SD 18.1], 66% male, mean body mass index [BMI] 26.3 [SD 5.18]) hospitalized at 62 intensive care units in 18 countries. Hospital mortality was 11% (n = 139), 44% (n = 536) were extubated by HD7 of which 20% (107/536) required reintubation, 28% (n = 340) underwent tracheostomy placement, and 9% (n = 114) developed ARDS. The median MP on HD1, HD3, and HD7 was 11.9 J/min [IQR 9.2–15.1], 13 J/min [IQR 10–17], and 14 J/min [IQR 11–20], respectively. MP was overall higher in patients with ARDS, especially those with higher ARDS severity. After controlling for same-day pressure of arterial oxygen/fraction of inspired oxygen (P/F ratio), BMI, and neurological severity, MP at HD1, HD3, and HD7 was independently associated with hospital mortality, reintubation and tracheostomy placement. The adjusted relative risk (aRR) was greater at higher MP, and strongest for: mortality on HD1 (compared to the HD1 median MP 11.9 J/min, aRR at 17 J/min was 1.22, 95% CI 1.14–1.30) and HD3 (1.38, 95% CI 1.23–1.53), reintubation on HD1 (1.64; 95% CI 1.57–1.72), and tracheostomy on HD7 (1.53; 95%CI 1.18–1.99). MP was associated with the development of moderate-severe ARDS on HD1 (2.07; 95% CI 1.56–2.78) and HD3 (1.76; 95% CI 1.41–2.22).

**Conclusions:**

Exposure to high MP during the first week of MV is associated with poor clinical outcomes in ABI, independent of P/F ratio and neurological severity. Potential benefits of optimizing ventilator settings to limit MP warrant further investigation.

**Supplementary Information:**

The online version contains supplementary material available at 10.1186/s13054-023-04410-z.

## Introduction

Patients with acute brain injury (ABI) commonly require intubation and mechanical ventilation (MV) due to insufficient airway protective reflexes, impaired respiratory drive, and secondary pulmonary events such as aspiration/pneumonia, pulmonary edema, or acute respiratory distress syndrome (ARDS) [1–6]. However, there are insufficient data to guide optimal ventilatory targets for brain injured patients [7]. Patients with ABI have frequently been excluded from landmark studies guiding MV practices in critical care cohorts [8–10], and the impact of various MV parameters on clinical outcomes is insufficiently explored in this population.

The concept of mechanical power (MP) as a determinant of ventilator-induced lung injury (VILI) has gained increasing attention [11]. A summary variable comprised of all the MV components which can cause VILI, including pressure, volume, flow, and respiratory rate (RR), MP is thought to represent the total energy delivered to the respiratory system during each breathing cycle multiplied by RR. Recent studies have demonstrated an association between higher MP and mortality in mixed critical care populations [12], both with [13] and without ARDS [14], and in patients with hypoxemic-ischemic encephalopathy (HIE) after cardiac arrest [15]. However, limited data are available regarding the role of MP in other ABI populations, with only one retrospective, single-center study showing an association between MP in the first 24 h and mortality during the intensive care unit (ICU) stay [16].

We performed a preplanned secondary analysis of the “Extubation strategies in neuro-intensive care unit patients and associations with outcomes (ENIO)” study—a prospective, international, multi-center observational cohort study assessing factors associated with extubation failure [17]. Our goals were to describe MP in ABI during the first week of MV and evaluate associations between MP at three time points and clinical outcomes. We hypothesized that there is substantial practice variation based on region and presence of ARDS, and that MP is associated with hospital mortality, need for reintubation, tracheostomy placement, and development of moderate-severe ARDS.

## Methods

*Setting* The present analysis utilized data from the ENIO study (NCT03400904), a prospective, international, multi-center observational cohort study that enrolled 1512 patients with ABI between 2018 and 2020 at 73 centers in 18 countries. The aims of the parent study were to describe current MV management and weaning practices, assess the incidence of extubation failure, rate of tracheostomy placement, and validate a score predictive of extubation success [17].

*Ethical approval and reporting* All centers were required to obtain regional or national IRB approval to participate in the ENIO study. The study was approved by the Steering Committee, no further IRB approval was necessary for the present analysis. We adhered to the Strengthening the Reporting of Observational Studies in Epidemiology (STROBE) guidelines [18].

*Study population* The ENIO study included adults (≥ 18 years) with ABI who were admitted to the intensive care unit (ICU) with a Glasgow Coma Scale (GCS) ≤ 12 before endotracheal intubation, and required invasive MV ≥ 24 h. Subtypes of brain injuries considered were traumatic brain injury (TBI), aneurysmal subarachnoid hemorrhage (SAH), intracranial hemorrhage (ICH), acute ischemic stroke (AIS), central nervous system infections, and brain tumors. Patients were excluded if they met any of the following criteria: < 18 years of age, pregnancy, spinal cord injury above T4, resuscitated after cardiac arrest, Guillain–Barre syndrome, withdrawal of life-sustaining treatments (WLST) ≤ 24 h after ICU admission, terminal extubation in the setting of WLST during ICU course, baseline major respiratory co-morbidities (requiring chronic oxygen at home, chronic obstructive pulmonary disease grade III-IV according to the Gold classification), and major chest trauma (Abbreviated Injury Score ≥ 3).

For the present analysis, we additionally excluded patients with insufficient data to calculate MP on hospital day (HD) 1, or those who were on a spontaneous breathing mode on HD1.

*Objectives* The primary aim of this study was to assess the use of MP on HD1, HD3, and HD7 in mechanically ventilated, brain-injured patients; our secondary aim was to evaluate the associations between MP at the three time points and clinical outcomes.

Clinical outcomes included hospital mortality- defined as death during the first hospital stay following ABI—need for reintubation during the initial ICU stay, tracheostomy placement during the initial ICU stay, and development of moderate-severe ARDS based on the Berlin definition [19] during the ICU stay.

*Data extraction* Demographic and baseline data were collected at the time of enrollment. Ventilatory parameters, arterial blood gas values, and use of sedative medications (Propofol, Midazolam, Dexmedetomidine, Penthotal), were recorded at HD1, HD3 and HD7 after ICU admission. Markers of neurological severity documented included: initial GCS before intubation, anisocoria, placement of an intracranial pressure (ICP) monitor or external ventricular drainage (EVD), decompressive craniectomy (DC), and barbiturate coma. Ventilatory parameters recorded for each HD included: tidal volume (V_t_), RR, positive end-expiratory pressure (PEEP), and plateau pressure (Pplat); also, the partial pressure of arterial oxygen/fraction of inspired oxygen (P/F) ratio and driving pressure (Δ*P*) were calculated for each HD. MP was calculated based on previously validated formulas [11, 20, 21]. Data on mortality, reintubation, tracheostomy and ARDS were prospectively captured during the index hospitalization, and follow-up was completed at hospital discharge.

*Statistical analysis* We summarized continuous variables using ranges, means (standard deviations, SD) or median (interquartile range, IQR) and categorical variables using percentages. Means, medians and frequencies were compared using the t-test, Wilcoxon-Mann–Whitney test, and chi-squares, respectively. We utilized multivariable log binomial regressions with robust standard errors to evaluate the associations between MP on HD1, HD3, and HD7 and (1) hospital mortality, (2) need for reintubation, (3) tracheostomy placement, and (4) development of moderate-severe ARDS. For development of moderate-severe ARDS, we assessed MP only on HD1 and HD3 as exposure variables, because  we were not able to discern which proportion of patients developed ARDS before HD7. We built separate models for each outcome of interest, adjusting for baseline characteristics, comorbidities, body mass index (BMI), region, type of brain injury, markers of neurological severity, and arterial blood gas values (Additional file 1: Appendix). A priori confounders and variables that were statistically significant in univariate analyses (P < 0.05) were considered for inclusion in multivariable analyses. The linearity assumption of continuous variables was tested, and variable transformed with the appropriate fractional polynomials when the assumption was not met [22]. Adjusted risk ratios (aRR) were calculated in comparison to the median MP utilized on HD1. We utilized R Studio 2022.02.3 and Stata 15.1 for statistical analyses.

## Results

### Patient characteristics

Of 1512 patients enrolled in the ENIO study, we excluded 286 (17%) patients due to insufficient data to calculate MP, and another 9 (0.6%) who were on a spontaneous breathing mode on HD1 (Additional file 1: Figure S1). We included 1217 patients from 18 countries at 62 centers in the analysis; the mean age was 51 years (SD 18), the majority were male (66%, n = 805), and mean BMI was 26.3 (SD 5.18). Most patients were from Europe/Central Asia (69%, n = 845) or Latin America/Caribbean (19%, n = 236). The most common underlying diagnoses were TBI (48%, n = 588), ICH (31%, n = 382), and SAH (18%, n = 218). The initial GCS before intubation was ≤ 8 in 77% (n = 937), 28% (n = 338) had an episode of anisocoria during their hospitalization (Table 1).

**Table 1:** Patient characteristics: demographics, comorbidities, baseline and clinical characteristics by hospital mortality

	Survivors	Non-survivors	Total
	(N = 1034)	(N = 139)	(N = 1217)*
*Baseline characteristics*			
Age (years)†	50.0 (17.9)	60.2 (17.4)	51.2 (18.1)
Female	346 (33%)	52 (37%)	412 (34%)
Height (cm)†	170 (9.17)	169 (9.28)	170 (9.18)
Weight (kg)†	76.4 (16.1)	73.1 (16.7)	76.0 (16.2)
BMI (cm/kg)†	26.3 (5.14)	25.7 (5.52)	26.3 (5.18)
*Geographic region*			
Europe and Central Asia	718 (69%)	97 (70%)	845 (69%)
Latin America & Caribbean	218 (21%)	14 (10%)	236 (19%)
South Asia	44 (4%)	22 (16%)	75 (6%)
Middle East and North Africa	23 (2%)	1 (1%)	25 (2%)
East Asia and Pacific	24 (2%)	5 (4%)	29 (2%)
North America	7 (1%)	0 (0%)	7 (1%)
*Country Income Level†††*			
High	792 (77%)	106 (76%)	928 (76%)
Upper middle	198 (19%)	11 (8%)	214 (18%)
Lower middle	44 (4%)	22 (16%)	75 (6%)
*Comorbidities*			
Hypertension	274 (27%)	56 (40%)	346 (28%)
Diabetes	110 (11%)	30 (22%)	146 (12%)
Heart failure	22 (2%)	11 (8%)	35 (3%)
Pulmonary disease	43 (4%)	2 (1%)	48 (4%)
Malignancy	40 (4%)	10 (7%)	53 (4%)
Current tobacco use	241 (23%)	25 (18%)	280 (23%)
*Clinical characteristics*			
Type of brain injury			
Traumatic brain injury	518 (50%)	47 (34%)	588 (48%)
Subarachnoid hemorrhage	177 (17%)	30 (22%)	218 (18%)
Intracranial hemorrhage	317 (31%)	50 (36%)	382 (31%)
Acute ischemic stroke	78 (8%)	17 (12%)	97 (8%)
CNS infection	45 (4%)	13 (9%)	60 (5%)
Brain tumor	52 (5%)	7 (5%)	60 (5%)
Initial GCS††	7.00 [3.00, 12.0]	7.00 [3.00, 12.0]	7.00 [3.00, 12.0]
GCS eyes††	1.00 [1.00, 4.00]	1.00 [1.00, 4.00]	1.00 [1.00, 4.00]
GCS verbal††	1.00 [1.00, 4.00]	1.00 [1.00, 4.00]	1.00 [1.00, 4.00]
GCS motor††	4.00 [1.00, 6.00]	4.00 [1.00, 6.00]	4.00 [1.00, 6.00]
Anisocoria	286 (28%)	42 (30%)	338 (28%)
Posterior fossa injury	57 (6%)	10 (7%)	70 (6%)
Nosocomial VAP	406 (39%)	61 (44%)	480 (40%)
ARDS	93 (9%)	19 (14%)	114 (9%)
Mild	19 (20%)	4 (21%)	24 (21%)
Moderate	36 (39%)	6 (32%)	43 (38%)
Severe	38 (41%)	9 (47%)	47 (41%)
*Treatment modalities utilized*			
Intraparenchymal ICP monitor	454 (44%)	35 (25%)	504 (41%)
Extraventricular drain	298 (29%)	52 (37%)	360 (30%)
Decompressive craniectomy	175 (17%)	32 (23%)	218 (18%)
Intracranial neurosurgery	403 (39%)	57 (41%)	483 (40%)
Barbiturate coma	65 (6%)	5 (4%)	74 (6%)
Therapeutic hypothermia	51 (5%)	5 (4%)	56 (5%)

Clinical characteristics based on hospital mortality

*Data on hospital mortality was missing in 44 patients

ARDS = acute respiratory distress syndrome, BMI = body mass index, CNS = central nervous system, GCS = Glasgow Coma Scale, ICP = intracranial pressure, VAP = ventilator associated pneumonia

†Mean (standard deviation)

††Median (interquartile range)

†††The Country Income Level was based on the World Health Organization

Mortality at ICU discharge was 7% (n = 83), and increased to 11% (n = 139) by hospital discharge. WLST occurred in 29% (n = 41) of all hospital mortalities at a median of 17 days [IQR 7–34]. Among survivors, 34% (356/1034) had a GCS of 13–15 at the time of extubation. By HD3, 23% (n = 270) of patients were extubated and 8% (n = 101) were weaned to a spontaneous mode; by HD7, 44% (n = 536) were extubated and 10% (n = 120) were weaned to a spontaneous mode (Additional file 1: Figure S2). Among patients who were extubated by HD7, 20% (107/536) required reintubation. Among patients who underwent tracheostomy placement (28%, n = 340), most (79% n = 267/340) received a tracheostomy without a prior extubation trial. Overall, 9% (n = 114) developed ARDS (21% (n = 24) mild, 38% (n = 43) moderate, and 41% (n = 47) severe ARDS).

Ventilator settings and arterial blood gas values by HD are summarized in Table 2. Baseline characteristics and markers of disease severity stratified by HD are displayed in Additional file 1: Fig. S3, Table S1, and were largely similar across the time points.

**Table 2:** Ventilator settings and respiratory parameters by hospital day

	Day 1	Day 3	Day 7
	N = 1216	N = 1075	N = 748
*Ventilator mode*			
Volume control	881 (72%)	558 (52%)	288 (39%)
Pressure control	335 (28%)	416 (39%)	340 (45%)
Spontaneous breathing	0	101 (9%)	120 (16%)
Tidal volume, mL/kg PBW†	7.14 [6.36;8.07]	7.21 [6.39;8.26]	7.38 [6.50;8.50]
Plateau pressure, cm H_2_O†	16 [14;19]	16 [14;20]	17 [14;20.5]
PEEP, cm H_2_O†	5 [5;6]	6 [5;7]	6 [5;8]
Driving pressure, cm H_2_O†	10 [8;13]	10 [8;13]	10 [7;13]
Respiratory rate, breaths/min†	16 [14;18]	16 [14;20]	18 [15;22]
Mechanical Power, Joules/min	11.9 [9.17;15.1]	12.6 [9.6;16.7]	14.3 [10.5;19.6]
PaO_2_, mmHg^†^	116 [91;158]	99 [83.2;121]	95 [81;114]
PaCO_2_, mmHg^†^	37 [34;41]	38 [35;41]	38 [34;42]

*IQR* interquartile range, PaCO_2_ partial arterial pressure of carbon dioxide, PaO_2_ partial arterial pressure of oxygen, *PEEP* positive end-expiratory pressure, *PBW* predicted body weight

^†^The daggers highlight which data is presented as mean vs median, and then how the World Income Level was defined

### Utilization of mechanical power and trajectories

The median MP on HD1, HD3, and HD7 was 11.9 J/min [IQR 9.2–15.1, range 3.1–44.4], 13 J/min [IQR 10–17, range 2.5–39.2], and 14 J/min [IQR 11–20, range 3.5–66.9] respectively. Across countries, median MP ranged from 8.9 to 18.2 J/min (HD 1), 7.2–17.6 J/min (HD3), and 8.3–19.2 J/min (HD7) (Additional file 1: Table S2, Figure S4). MP mainly varied based on the presence of ARDS, was higher in patients with higher ARDS severity, and did not substantially differ based on neurological severity (Table 3). MP on HD3 and HD7 was significantly higher in patients on sedative medications, compared to those who did not receive any sedation (Table 4).

**Table 3:** Mechanical power at hospital day one, three, and seven

	Hospital day 1	Hospital day 3	Hospital day 7
	N = 1216	N = 1075	N = 748
Overall	11.9 (9.2;15.1)	13 (10;17)	14 (11;20)
*Region*			
Europe & Central Asia	11.9 (9.3;15.3)	12.6 (9.7;17.3)	15 (11;21)
Latin America & Caribbean	11.6 (8.8;14.6)	12.5 (9.9;16.0)	14.1 (10.8;17.6)
South Asia	12.2 (9.5;15.4)	12.6 (9.5–16.5)	13.5 (8.9;17.5)
Middle East and North Africa	12.5 (8.9;17.6)	11.0 (8.6;16.4)	8.4 (7.4;10.4)
East Asia and Pacific	11.1 (7.8;14.5)	11.1 (8.6;15.3)	13.6 (11.4;15.5)
North America	15.0 (13.9;16.1)	15.0 (11.8;15.5)	19.2 (15.2;23.2)
*Country income level*			
High	11.9 (9.3;15.3)	12.7 (9.8;17.2)	15 (11;21)
Upper middle	11.4 (8.8;14.6)	11.9 (8.9;15.7)	13.3 (9.1;16.9)
Lower middle	12.2 (9.5;15.4)	12.6 (9.5;16.5)	13.5 (8.9;17.5)
Low	–	–	–
*By ventilator mode*			
Volume control	11.8 (9.2;14.8)	12.7 (9.9; 16.9)	15.7 (11.8; 21.5)
Pressure control	12.3 (9.2;16.3)	12.3 (8.9; 16.5)	13.2 (9.8; 17.5)
*By type of brain injury*			
Traumatic brain injury	12.2 (9.4;15.4)	12.7 (10;17)	15 (11;22)
Subarachnoid hemorrhage	11.2 (8.7;14.7)	12.8 (9.5;17.7)	16 (11.9;20.7)
Intracranial hemorrhage	12.3 (9.5;15.6)	12.4 (9.9;16.2)	13.3 (10.0;17.5)
Acute Ischemic Stroke	10.7 (8.8;14.0)	12 (8.8;15.3)	13.1 (10.3;17.4)
CNS infection	11.1 (8.7;15.0)	11.5 (8.2;14.3)	11.6 (8.1;15.8)
Brain Tumor	10.5 (8.2;12.6)	10.8 (7.6;15.2)	11.9 (7.5;15.6)
*GCS on admission*			
3–8	11.9 (9.2;15.1)	12.4 (9.6;16.4)	14 (10;19)
9–12	12.1 (9.2;15.2)	13.6 (9.8;17.4)	15 (11.8;20.3)
13–15	–	–	–
*ICP monitor placed*			
Yes	12.2 (9.4;15.5)	12.9 (9.9–17.6)	15.7 (11.2–22.8)
No	11.5 (9.0;15.0)	12.3 (9.4;16.0)	13.0 (10.0;18.0)
*Decompressive craniectomy*			
Yes	11.3 (8.7;14.4)	12.5 (9.5;16.1)	13.8 (10.1;18.6)
No	11.9 (9.3;15.4)	12.6 (9.7;16.9)	14.0 (11.0;20.0)
*ARDS*			
No ARDS	11.8 (9.0–14.8)	12.3 (9.5–16.0)	13.6 (10.0–17.9)
Mild	12.1 (9.7;16.8)	13.0 (8.8;16.1)	15.3 (13.8;21.2)
Moderate	15.5 (11.3;19.7)	17.1 (12.6;22.1)	18.4 (13.3;24.5)
Severe	14.0 (11.0;17.0)	17.2 (11.5;23.8)	23.8 (17.4;28.1)

*J/minute

Values reported in median (Interquartile range) *ARDS* acute respiratory distress syndrome, *CNS* central nervous system, *GCS* Glasgow Coma Scale, *ICP* intracranial pressure

**Table 4:** Mechanical power based on sedation strategy

Hospital day 1			Hospital day 3			Hospital day 7		
Sedation* (N = 1100)	No sedation (N = 116)	P	Sedation* (N = 733)	No sedation (N = 211)	P	Sedation* (N = 376)	No sedation (N = 279)	P
MP 12.0 (9.2;15.4)	MP 11.2 (9.0; 13.9)	0.062	MP 13.0 (10.1; 17.3)	MP 11.5 (8.7; 15.0)	0.0003	MP 15.5 (11.6; 21.4)	MP 13.8 (9.9; 17.8)	0.001

*MP* mechanial power

*Propofol, and/or Midazolam, and/or Dexmedetomidine, and/or Penthotal

**Fig. 1:**
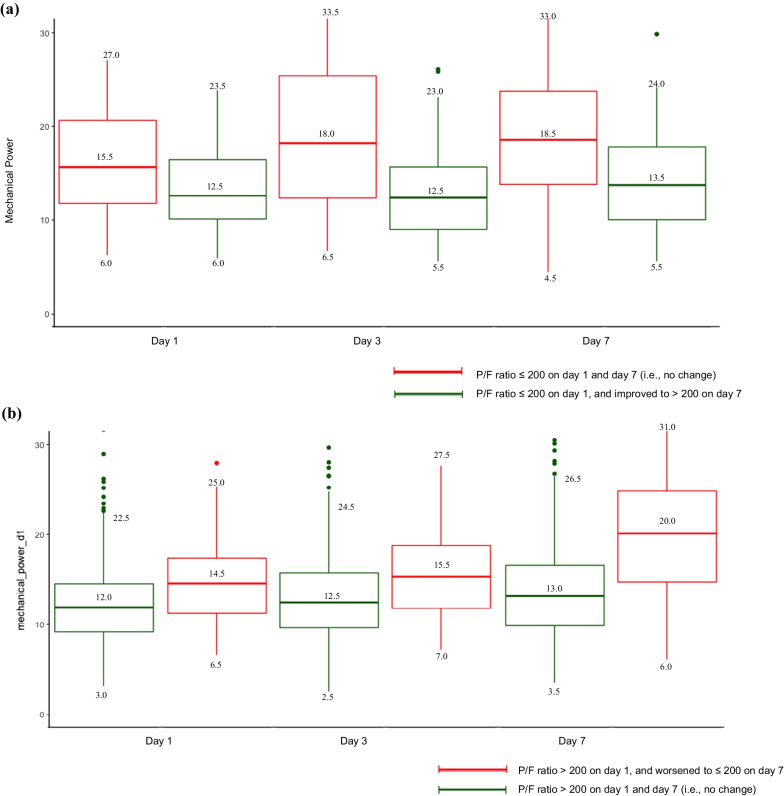

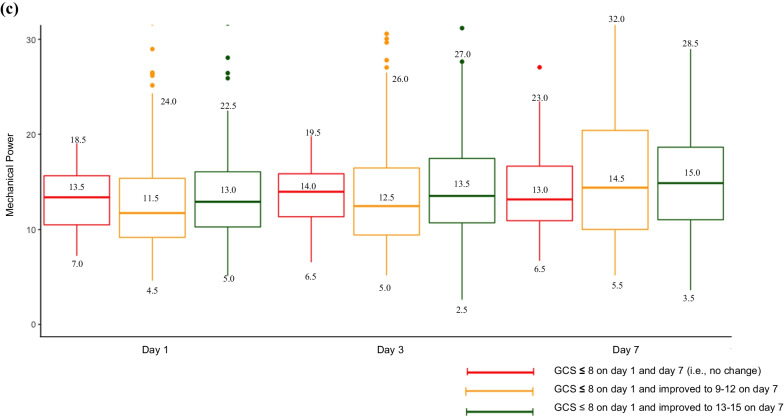
Mechanical power trajectories. **a** Mechanical power based on trajectories of P/F ratio for patients with P/F ratio ≤ 200 on hospital day 1. **b** Mechanical power based on trajectories of P/F ratio for patients with P/F ratio > 200 on hospital day 1. **c** Mechanical power based on trajectories of GCS for patients with an initial GCS ≤ 8

Median MP was higher on HD1, HD3 and HD7 among patients with an initial P/F ratio ≤ 200 and whose P/F ratio remained ≤ 200 compared to those whose P/F ratio improved to > 200 by HD7 (Fig. 1a). Additionally, median MP was higher on HD1, HD3 and HD7 in patients with an initial P/F ratio > 200 whose P/F ratio worsened to ≤ 200 by HD7, compared to those whose P/F ratio remained > 200 by HD7 (Fig. 1b). Among patients with an initial GCS ≤ 8, the median MP on HD1, HD3 and HD7 was largely similar in patients whose GCS remained ≤ 8 and those whose GCS improved by the time of extubation (Fig. 1c).

### Associations between mechanical power and clinical outcomes

**Fig. 2:**
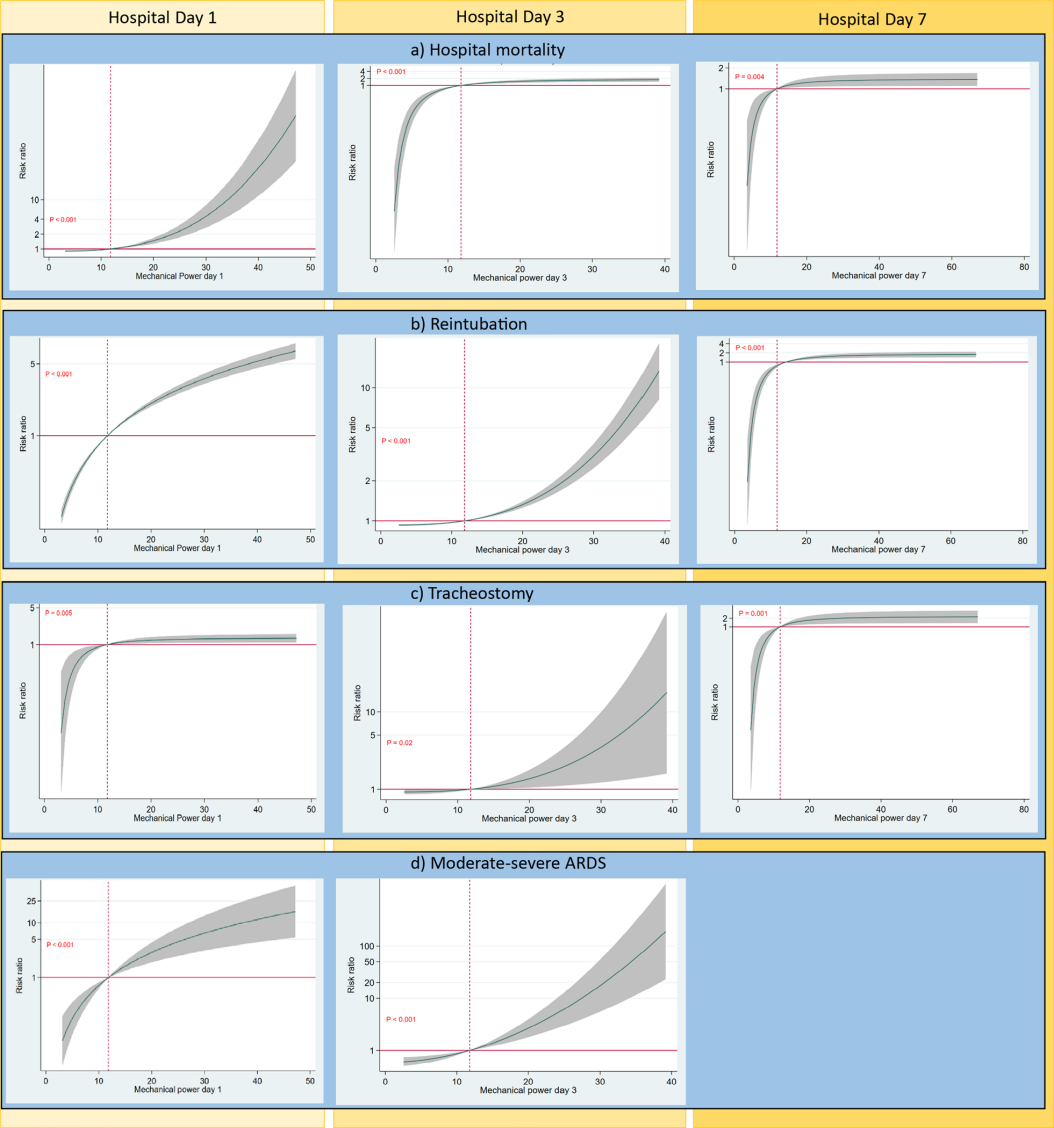
Associations between mechanical power (MP) and clinical outcomes at hospital day one, three and seven. EVD = Extraventricular Drain, GCS = Glasgow Coma Scale, ICP = Intracranial Pressure. **a** Associations between MP and mortality. The model was adjusted for the following covariates: age, region, comorbidities (heart failure, diabetes, pulmonary disease), body mass index, type of brain injury, neurological severity (initial GCS, anisocoria, ICP monitor in place, EVD in place, decompressive craniectomy, barbiturate coma), arterial blood gas values (same day P/F ratio), ventilator mode, and sedation (same day Propofol, Midazolam). **b** Associations between MP and need for reintubation. The model was adjusted for the following covariates: age, region, comorbidities (heart failure, hypertension, pulmonary disease), body mass index, type of brain injury, neurological severity (initial GCS, anisocoria, ICP monitor in place, EVD in place, decompressive craniectomy, barbiturate coma), swallowing function on the day of extubation, suctioning frequency on the day of extubation,, arterial blood gas values (same day P/F ratio), ventilator mode, and sedation (same day Propofol, Midazolam). **c** Associations between MP and tracheostomy placement. The model was adjusted for the following covariates: age, region, comorbidities (heart failure, hypertension pulmonary disease), body mass index, type of brain injury, neurological severity (initial GCS, anisocoria, ICP monitor in place, EVD in place, decompressive craniectomy, barbiturate coma), swallowing function on the day of extubation, suctioning frequency on the day of extubation,, arterial blood gas values (same day P/F ratio), ventilator mode, and sedation (same day Propofol, Midazolam). **d** Associations between MP and development of moderate to severe acute respiratory distress syndrome (ARDS). The model was adjusted for the following covariates: age, sex, region, baseline pulmonary or cardiac disease, type of brain injury, initial GCS, decompressive craniectomy, arterial blood gas values (same day PaO_2_ and PaCO_2_), ventilator mode and ventilator associated pneumonia

In separate multivariable models controlling for age, BMI, region, comorbidities, type of brain injury, neurological severity, same day P/F ratio, ventilator mode and sedation, MP on HD1, HD3, and HD7 was independently associated with hospital mortality, with greater aRR at higher MP (Fig. 2a, Additional file 1: Table S3a, omnibus p-values for non-linear trajectories were p < 0.001, p < 0.001, p = 0.003 respectively). Within the most commonly utilized range of 9–20 J/min, the aRR (compared to the HD1 median of 11.9 J/min) for HD1 was 1.22 (95% CI 1.14–1.30) at 17 J/min and 1.47 (95% CI 1.28–1.65) at 20 J/min. On HD3, the aRR was 1.38 (95% CI 1.23–1.53) at 17 J/min and 1.49 (95% CI 1.30–1.71) at 20 J/min, and on HD7, the aRR was 1.06 (95% CI 1.05–1.32) at 17 J/min and 1.23 (95% CI 1.07–1.42) at 20 J/min. (Table 5) The increment in aRR for each J/min was highest on HD1.

**Table 5:** Adjusted relative risks of clinical outcomes associated with mechanical power (aRR were calculated compared to median on HD 1 of 11.85 Joules/min): (a) mortality, (b) need for reintubation, (c) tracheostomy placement, (d) development of moderate to severe acute respiratory distress syndrome (ARDS)

Mechanical power, J/min	aRR (95% CI)		
	Day 1	Day 3	Day 7
*(a) Hospital mortality*			
12	1.01 (1.00–1.01)	1.02 (1.01–1.03)	1.01 (1.00–1.02)
13	1.03 (1.02–1.05)	1.12 (1.07–1.16)	1.06 (1.02–1.09)
14	1.07 (1.04–1.09)	1.20 (1.13–1.27)	1.10 (1.03–1.17)
15	1.11 (1.07–1.15)	1.26 (1.17–1.37)	1.13 (1.04–1.23)
16	1.16 (1.10–1.22)	1.32 (1.20–1.46)	1.16 (1.05–1.28)
17	1.22 (1.14–1.30)	1.38 (1.23–1.53)	1.06 (1.05–1.32)
18	1.29 (1.18–1.40)	1.42 (1.26–1.60)	1.20 (1.06–1.36)
19	1.37 (1.23–1.53)	1.46 (1.28–1.66)	1.22 (1.07–1.39)
20	1.47 (1.28–1.65)	1.49 (1.30–1.71)	1.23 (1.07–1.42)
21	1.58 (1.35–1.85)	1.52 (1.32–1.76)	1.25 (1.07–1.45)
*(b) Reintubation*			
12	1.03 (1.03–1.03)	1.01 (1.00–1.01)	1.00 (1.00–1.00)
13	1.15 (1.13–1.16)	1.02 (1.02–1.03)	1.03 (1.02–1.03)
14	1.26 (1.24–1.29)	1.05 (1.04–1.06)	1.05 (1.04–1.06)
15	1.39 (1.35–1.43)	1.08 (1.06–1.09)	1.06 (1.04–1.08)
16	1.52 (1.46–1.57)	1.11 (1.09–1.14)	1.13 (1.08–1.18)
17	1.64 (1.57–1.72)	1.15 (1.12–1.19)	1.19 (1.12–1.26)
18	1.78 (1.69–1.88)	1.20 (1.16–1.25)	1.25 (1.15–1.35)
19	1.92 (1.81–2.03)	1.26 (1.21–1.31)	1.29 (1.18–1.42)
20	2.05 (1.92–2.20)	1.32 (1.25–1.39)	1.34 (1.20–1.48)
21	2.20 (2.05–2.36)	1.40 (1.31–1.49)	1.37 (1.22–1.53)
*(c) Tracheostomy placement*			
12	1.01 (1.00–1.02)	1.01 (1.00–1.01)	1.02 (1.01–1.03)
13	1.05 (1.02–1.09)	1.03 (1.00–1.05)	1.17 (1.06–1.29)
14	1.09 (1.03–1.15)	1.06 (1.01–1.11)	1.27 (1.10–1.47)
15	1.12 (1.03–1.21)	1.09 (1.01–1.17)	1.37 (1.13–1.67)
16	1.14 (1.04–1.25)	1.13 (1.02–1.25)	1.46 (1.16–1.84)
17	1.16 (1.05–1.29)	1.17 (1.03–1.34)	1.53 (1.18–1.99)
18	1.18 (1.05–1.33)	1.23 (1.03–1.46)	1.60 (1.20–2.15)
19	1.20 (1.06–1.35)	1.29 (1.04–1.60)	1.66 (1.22–2.26)
20	1.21 (1.06–1.39)	1.36 (1.05–1.77)	1.71 (1.23–2.39)
21	1.22 (1.06–1.40)	1.45 (1.06–1.99)	1.75 (1.24–2.48)
*(d) ARDS*			
12	1.04 (1.02–1.06)	1.03 (1.02–1.04)	
13	1.22 (1.13–1.32)	1.12 (1.07–1.17)	
14	1.41 (1.23–1.61)	1.24 (1.14–1.36)	
15	1.62 (1.34–1.95)	1.38 (1.21–1.57)	
16	1.84 (1.45–2.33)	1.55 (1.30–1.85)	
17	2.07 (1.56–2.78)	1.76 (1.41–2.22)	
18	2.33 (1.67–3.24)	2.01 (1.52–2.66)	
19	2.58 (1.78–3.78)	2.30 (1.65–3.21)	
20	2.88 (1.89–4.27)	2.64 (1.79–3.90)	
21	3.17 (2.02–4.97)	3.10 (1.97–4.89)	

MP on all three hospital days was associated with need for reintubation after controlling for covariates, including factors associated with extubation failure in the main ENIO analysis (Fig. 2b, Additional file 1: Table S3b, omnibus p-values for non-linear trajectories were all < 0.001). Compared to the HD1 median, the aRR was strongest on HD1 :1.64 (95% CI 1.57–1.72) at 17 J/min and 2.05 (95% CI 1.92–2.20) at 20 J/min) and the increment in aRR for each J/min was steepest on HD1 and HD3. (Table 5).

MP on all three hospital days was associated with tracheostomy placement after controlling for covariates (Fig. 2c, Additional file 1: Table S3c, omnibus p-values for non-linear trajectories were p = 0.005, p = 0.019, and p = 0.001, respectively); the aRR was strongest on HD7:1.53 (95% CI 1.18–1.99) at 17 J/min and 1.71 (95% CI 1.23–2.39) at 20 J/min (Table 5).

MP on HD1 and HD3 was associated with moderate-severe ARDS after controlling for covariates (Fig. 2d, Table S3d, omnibus p-values for non-linear trajectories were p < 0.001 for both days). For HD1, aRR compared to the HD1 median was 2.07 (1.56–2.78) at 17 J/min and 2.88 (1.89–4.27) at 20 J/min; for HD3, aRR was 1.76 (1.41–2.22) at 17 J/min and 2.64 (1.79–3.90) at 20 J/min (Table 5).

## Discussion

In this preplanned, secondary analysis of the ENIO study assessing use and effect of mechanical power in ABI, our main findings are: (1) MP varied widely by region, tended to be higher based on presence and severity of ARDS, and differed by trajectory of P/F ratio, (2) MP at all time points was associated with hospital mortality, need for reintubation, tracheostomy placement and development of moderate-severe ARDS, and (3) associations between MP and hospital mortality, reintubation, and ARDS were strongest during the early days of MV. To date, this is the largest observational investigation evaluating the use of MP in ABI and its association with clinical outcomes. We included patients from 62 institutions in 18 countries, representing current practices worldwide.

In critically ill patients with ABI, pathophysiological interactions between brain and lungs are complex and bi-directional [6]. ABI has been shown to induce or worsen lung injury via several mechanisms, including elevated ICP, systemic inflammatory response, hormonal dysregulation, and catecholamine surges [6, 23, 24]. Conversely, hypoxemia and systemic inflammation can precipitate secondary brain injury, and ventilatory strategies that have proven to be beneficial for management of ARDS, such as PEEP titration, low V_t_, and prone ventilation, may affect ICP, and cerebral perfusion pressure. [6, 25, 26]. As ABI comprises up to 20% of patients requiring MV [27, 28], with little data to guide ventilator management accounting for brain-lung crosstalk [29], more information about use and effect of MV strategies is important to inform future studies, and establish optimal ventilatory targets for this population.

The substantial regional practice variations in utilization of MP in our study underscore the lack of definite knowledge and data on ventilator management in ABI. Results of a large, prospective multicenter observational study (NCT04459884) are forthcoming [30], and randomized controlled trials (RCTs) are needed to provide more clarity on this topic.

Overall, medians and ranges of MP observed in our study were similar to values described in another ABI cohort [16], but lower compared to those described in a large general critical care cohort [12] and a post-cardiac arrest cohort [15], possibly due to higher proportions of lung injury in these populations. Our results also suggest that MP utilization is mainly driven by concomitant lung injury and ARDS. The question of whether markers of neurological severity merit consideration in choosing ventilator settings warrants further exploration.

The associations between MP and clinical outcomes in the first week of mechanical ventilation, present as early as HD1, support the hypothesis that MV settings precipitate VILI. Different ventilator variables (V_t_, [8, 31] Pplat [8], PEEP [32, 33], driving pressure [34–36]) have been associated with mortality and longer duration of MV, presumably by contributing to VILI. Strategies targeted at minimizing VILI, specifically the prophylactic utilization of low V_t_, [31, 37–39], have shown promise in decreasing the risk of developing ARDS. However, titrating one single MV setting may not adequately protect the lungs if the total amount of total energy delivered to the lungs is similar [11]. MP, reflecting the combined effect of various MV settings, has recently emerged as a real-life marker of VILI and predictor of clinical outcomes [11–13, 16, 30, 40, 41].

Experimental research has linked MP to diffuse radiographic pulmonary edema, and increased in lung elastance [40], and has suggested correlations between MP and pulmonary neutrophilic inflammation [41]. A study including 8207 critically ill patients requiring MV for > 48 h showed an association between MP at 24–48 h and 30-day mortality, even at low V_t_ and driving pressure, and independent of ARDS [12]. A combined analysis of 4549 patients with ARDS demonstrated an association between MP within 24 h and 28-day mortality [13]. A recent, combined analysis of three RCTs also found an association between MP and mortality in non-ARDS populations, even after stratifying for individual components of MP [14]. In the cardiac arrest population, a sub-analysis of the targeted hypothermia versus targeted normothermia after out-of-hospital cardiac arrest (TTM-2) trial including 1848 patients with HIE showed an association between MP in the first 72 h of MV and 6-months mortality [15]. In other populations with ABI, only one retrospective study assessing 529 brain-injured patients demonstrated an association between MP in the first 24 h and ICU mortality, with MP being a stronger predictor of mortality compared to GCS [16].

Our findings are consistent with previous studies and complement this data by showing associations between MP and mortality in a large prospective cohort of ABI, measured at three different time points, controlling for BMI and same day P/F ratio. The associations in the early phases of MV, notable as early as HD1, suggest that there may be a critical time window during which MP could exert a deleterious effect. Also, the associations of MP with moderate-severe ARDS on HD1 and HD3 suggest that MP may be linked to poor outcomes by contributing to VILI. Additionally, we found that MP is associated with reintubation and tracheostomy placement. Our ability to predict which patients with ABI can be extubated remains limited [17, 42]. Recent studies have identified predictors of successful extubation, such as eye movements, gag, cough, secretion burden and GCS [17, 43–45]. To our knowledge, this is the first study showing an association between ventilatory settings and reintubation or tracheostomy placement in brain-injured patients. Of note, the majority of patients in our study who underwent tracheostomy placement did not have an extubation trial, likely because providers had higher concerns about their ability to wean from MV and tended to favor tracheostomy placement in those requiring more concerning ventilator settings. More interestingly, the association between MP and reintubation after adjusting for other factors associated with extubation failure in the main ENIO analysis, warrants further investigation. Early injurious effect of MV settings may render the lungs more vulnerable to subsequent ‘second hit-events’, such as aspiration or pneumonia, or contribute to pulmonary edema post extubation.

While difficult to directly compare results due to differences in methodology, our findings suggest a consistent increase in risk of death at lower thresholds (13 J/min) compared to that identified in a general critical care population (17 J/min) [12]. These findings indicate that patients with ABI may be more prone to VILI and other potential detrimental effects of MV, possibly due to brain-lung crosstalk.

Our study has several limitations. First, this is an observational study, which limits conclusions regarding causality. MP is ultimately a composite of parameters that are thought to cause VILI but are also linked to higher disease severity at the same time. While we controlled for markers of disease severity and arterial blood gas values, additional aspects of the patients’ underlying condition and systemic illness may have contributed to ventilator settings and higher MP in patients with worse clinical outcomes. Therefore, we cannot rule out residual confounding or confounding by indication. Second, we assessed the associations between MP and clinical outcomes separately at three time points; we did not have data on MP during the entire period of MV and therefore could not assess the cumulative impact of MP on clinical outcomes. Additionally, since MP on HD1 may be correlated with high MP on subsequent days, it is not possible to determine whether the associations on HD1 are due to an early injurious effect or related to subsequently high MP levels and cumulative lung injury. Third, our sample size decreased over time; while measured clinical characteristics across the time points are similar, the sample may differ by unmeasured covariates, so comparisons across time points are limited. Additionally, due to the smaller sample size at the later time points, some confidence intervals for associations on HD3 and HD7 are wide, limiting our ability to draw conclusions. Forth, we had insufficient power to assess how associations between MP and clinical outcomes vary by ABI subtype due low sample size within each category. Given distinct underlying pathophysiologies for different ABI subtypes, further research is needed to investigate if the impact of MV varies between disease entities. Last, based on the data available, we could not discern how many patients demonstrated a spontaneous respiratory drive while on a controlled mode, and how their own respiratory efforts may have affected MP.

Despite these limitations, our findings can serve as groundwork for future research evaluating the impact of limiting exposure to high MP, cumulative impact of exposure to MP during MV, and assessing injurious MV thresholds in ABI compared to other critical care cohorts. Future studies are also needed to examine how different components of the MP formula contribute to clinical outcomes, including conducting risk prediction modeling [46]. Also, studies collecting more frequent measurements during MV are needed to assess the time varying exposure of MP controlling for time varying confounders such as sedation or hemodynamic data, potentially using G-estimation or other advanced epidemiologic methods [47]. Ultimately, randomized clinical studies are required to assess a causal relationship between MP and clinical outcomes.

## Conclusions

Optimizing ventilator settings and limiting exposure to high MP during the first week of mechanical ventilation may be associated with better clinical outcomes in patients with ABI.

## Supplementary Information

**Additional file 1**. Appendix.

## Data Availability

The datasets used and/or analyzed during the current study are available from the corresponding author on reasonable request.

## Abbreviations

ABI: Acute brain injury
AIS: Acute ischemic stroke
ARDS: Acute respiratory distress syndrome
BMI: Body Mass Index
ENIO: Extubation strategies in neuro-intensive care unit patients and associations with outcomes
EVD: Extraventricular drain
DC: Decompressive craniectomy
GCS: Glasgow Coma Scale
HD: Hospital day
HIE: Hypoxemic-ischemic encephalopathy
ICH: Intracranial hemorrhage
ICP: Intracranial pressure
ICU: Intensive care unit
IQR: Interquartile range
MP: Mechanical power
MV: Mechanical ventilation
Δ*P*: Driving pressure
PEEP: Positive end-expiratory pressure
P/F: Partial pressure of arterial oxygen/fraction of inspired oxygen
P Plat: Plateau pressure
RCT: Randomized controlled trial
RR: Respiratory rate
SAH: Subarachnoid hemorrhage
SD: Standard deviation
TBI: Traumatic brain injury
VILI: Ventilator induced lung injury
Vt: Tidal volume
WLST: Withdrawal of life-sustaining treatments
