# Supplementary material for: Utilization of mechanical power and associations with clinical outcomes in brain injured patients: a secondary analysis of the extubation strategies in neuro-intensive care unit patients and associations with outcome (ENIO) trial
Source: Crit Care. 2023 Apr 20;27:156. doi: 10.1186/s13054-023-04410-z (PMC10120226; doi:10.1186/s13054-023-04410-z)
Supplement: Supplementary file 1 — Additional file 1. Appendix. [file 13054_2023_4410_MOESM1_ESM.docx]

**Supplementary appendix**

**Accompanying the manuscript:**

**Utilization of Mechanical Power and Associations with Clinical Outcomes in Brain Injured Patients: A secondary analysis of the Extubation Strategies in Neuro-Intensive Care Unit Patients and Associations with Outcome (ENIO) Trial**

Sarah Wahlster, MD FNCS^1,2,3*^, Monisha Sharma, PhD^4*^, Shaurya Taran, MD^5,6^, James A. Town, MD^7^, Robert D. Stevens, MD, PhD^8^, Raphaël Cinotti^9^, MD, Karim Asehoune, MD^9^, Paolo Pelosi, MD^10,11^, Chiara Robba, MD, PhD^10,11^

^1^ Department of Neurology, Harborview Medical Center, University of Washington, Seattle, USA

^2^ Department of Neurological Surgery, Harborview Medical Center, University of Washington, Seattle, USA

^3^ Department of Anesthesiology and Pain Medicine, Harborview Medical Center, University of Washington, Seattle, USA

^4^ Department of Global Health, University of Washington, Seattle, USA

^5^ Division of Respirology, Department of Medicine, University Health Network, Toronto, ON, Canada.

^6^ Interdepartmental Division of Critical Care Medicine, University of Toronto, Toronto, ON, Canada

^7^ Division of Pulmonary, Critical Care and Sleep Medicine, Department of Medicine, University of Washington, Seattle, USA

^8^ Department of Anesthesiology and Critical Care Medicine, Johns Hopkins University School of Medicine, Baltimore, MD, USA

^9^ Department of Anesthesiology and Critical Care, CHU Nantes, Nantes Université, Nantes, France

^10^ Department of Surgical Sciences and Integrated Diagnostics, University of Genoa, Genoa, Italy

^11^ Anesthesia and Critical Care, San Martino Policlinico Hospital, IRCCS for Oncology and Neurosciences, Genoa, Italy
*equal contributors

**Table of Contents**

**Figure S1:** Flow diagram of patients included in the analysis Page 3

**Figure S2:** Flow diagram of patients included in the multivariable analysis by hospital day Page 4

**Figure S3:** Baseline characteristics stratified by world region Page 5

**Figure S4:** Mechanical Power Stratified by Country, HD1 Page 6

**Table S1:** Baseline characteristics stratified by Hospital Day Page 7

**Table S2:** Mechanical power stratified by country (HD1, HD3, HD7) Page 9

**Table S3a:** Multivariable analyses, Hospital mortality Page 10

**Table S3b:** Multivariable analyses, Need for Reintubation Page 12

**Table S3c:** Multivariable analyses, Tracheostomy placement Page 14

**Table S3d:** Multivariable analyses, Moderate-severe ARDS Page 16

**Figure S1:** Flow diagram of patients excluded from the analysis

Patients enrolled in ENIO

(N = 1,512)

Insufficient data to

calculate MP (N=286)

Patients enrolled in ENIO,

sufficient data to calculate MP

(N = 1,226)

Spontaneous ventilator mode

on HD1 (N=9)

Patients included in the analysis

(N = 1,217)

ENIO= Extubation Strategies in Neuro-Intensive Care Unit Patients and Associations with Outcome, HD=hospital day, MP=mechanical power

**Figure S2:** Flow diagram of patients included in the multivariable analysis assessing associations between mechanical power and mortality

Patients included HD1

(N = 1,217)

Extubated**: N=254
Weaned to PSV: N=101

Missing Data****: N=146

Mortality/WLST: N=25/3

Patients included* HD3

(N = 688)

Extubated***: N=185
Weaned to PSV: N=19

Missing Data****: N=55

Mortality/WLST: N=37/11

Patients included* HD7

(N = 391)

HD=hospital day 1, PSV=pressure support ventilation
*patients included for the multivariable analysis assessing associations between mechanical power and mortality

** extubated after HD1, and remained extubated by HD3 (total extubated n=270)

*** extubated after HD 3, and remained extubated by HD7 (total extubated n=266)

**** insufficient data (ventilatory parameters) to calculate MP

**Figure S3: Baseline characteristics stratified by world region**


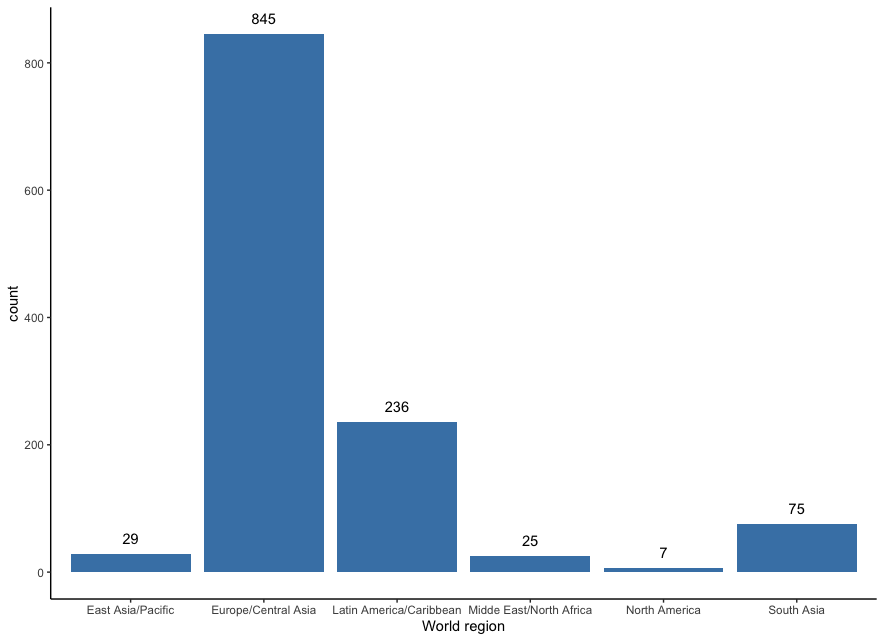


| **Characteristic** | **East Asia &**  **Pacific** | **Europe &**  **Central**  **Asia** | **Latin America &**  **Caribbean** | **Middle East &**  **North Africa** | **North America** | **South**  **Asia** |
| --- | --- | --- | --- | --- | --- | --- |
|  |  |  |  |  |  |  |
| **Age, median (IQR)** | 65 (53-71) | 58 (44-68) | 36 (26-52) | 59 (28-66) | 59 (59-59) | 37 (29-55) |
| **Female sex, n (%)** | 8 (27.6) | 318 (37.6) | 61 (25.8) | 2 (8.0) | 2 (28.6) | 21 (28.0) |
| **BMI, median (IQR)** | 22 (20-23) | 26 (23-29) | 26 (23-29) | 26 (22-29) | 32 (28-36) | 26 (24-27) |
| **Comorbidities, n (%)** |  |  |  |  |  |  |
| Hypertension | 12 (41.4) | 271 (32.1) | 36 (15.3) | 11 (44.0) | 5 (71.4) | 11 (14.7) |
| Diabetes | 5 (17.2) | 105 (12.4) | 19 (8.1) | 7 (28.0) | 2 (28.6) | 8 (10.7) |
| Heart failure | 0 (0.0) | 31 (3.7) | 2 (0.9) | 2 (8.0) | 0 (0.0) | 0 (0.0) |
| Pulmonary disease | 0 (0.0) | 43 (5.1) | 1 (0.4) | 3 (12.0) | 1 (14.3) | 0 (0.0) |
| Malignancy | 1 (3.4) | 47 (5.6) | 2 (0.9) | 2 (8.0) | 0 (0.0) | 1 (1.3) |
| Active tobacco use | 5 (17.2) | 201 (23.9) | 56 (23.8) | 8 (32.0) | 3 (42.8) | 7 (9.3) |
| **Brain injury diagnosis, n (%)** |  |  |  |  |  |  |
| Traumatic brain injury | 9 (31.0) | 353 (41.8) | 173 (73.3) | 9 (36.0) | 0 (0.0) | 44 (59.4) |
| Subarachnoid hemorrhage | 8 (27.6) | 190 (22.5) | 12 (5.1) | 0 (0.0) | 2 (28.6) | 6 (8.3) |
| Intracranial hemorrhage | 10 (34.4) | 270 (32.0) | 66 (28.0) | 17 (68.0) | 2 (28.6) | 17 (23.3) |
| Acute ischemic stroke | 3 (10.3) | 77 (9.1) | 9 (3.8) | 1 (4.0) | 3 (42.8) | 4 (5.6) |
| CNS infection | 1 (3.4) | 35 (4.1) | 14 (6.0) | 4 (16.0) | 1 (14.3) | 5 (6.9) |
| Brain tumor | 0 (0.0) | 43 (5.1) | 12 (5.2) | 0 (0.0) | 0 (0.0) | 5 (6.9) |
| **Clinical characteristics** |  |  |  |  |  |  |
| Lowest GCS before intubation,  median (IQR) | 6 (3-8) | 7 (4-8) | 7 (6-8) | 6 (3-8) | 8 (7-8) | 7 (5-9) |
| Anisocoria |  |  |  |  |  |  |
| Posterior fossa injury, n (%) | 0 (0.0) | 54 (6.4) | 14 (5.9) | 2 (8.0) | 0 (0.0) | 0 (0.0) |
| **ARDS** | 0 (0.0) | 90 (7.4) | 16 (1.3) | 3 (0.2) | 1 (0.1) | 4 (0.3) |
| Mild | 0 (0.0) | 18 (20.0) | 3 (18.8) | 1 (33.3) | 0 | 2 (50.0) |
| Moderate | 0 (0.0) | 30 (33.3) | 10 (62.4) | 2 (66.7) | 0 | 1 (25.0) |
| Severe | 0 (0.0) | 42 (46.7) | 3 (18.8) | 0 | 1 (100.0) | 1 (25.0) |
| **Treatment modalities, n (%)** |  |  |  |  |  |  |
| ICP monitor | 1 (3.4) | 458 (54.3) | 36 (15.2) | 1 (4.0) | 3 (42.8) | 5 (6.7) |
| External ventricular drain | 11 (37.9) | 315 (37.3) | 17 (7.2) | 4 (16.0) | 3 (42.8) | 10 (13.3) |
| Decompressive craniectomy | 1 (3.4) | 127 (15.0) | 55 (23.3) | 7 (28.0) | 1 (14.3) | 27 (36.0) |
| Barbiturate coma | 0 (0.0) | 42 (5.0) | 27 (11.4) | 1 (4.0) | 0 (0.0) | 4 (5.3) |
| Therapeutic hypothermia | 0 (0.0) | 55 (6.5) | 1 (0.4) | 0 (0.0 | 0 (0.0) | 0 (0.0) |

**Figure S4: Mechanical power stratified by country, HD1**


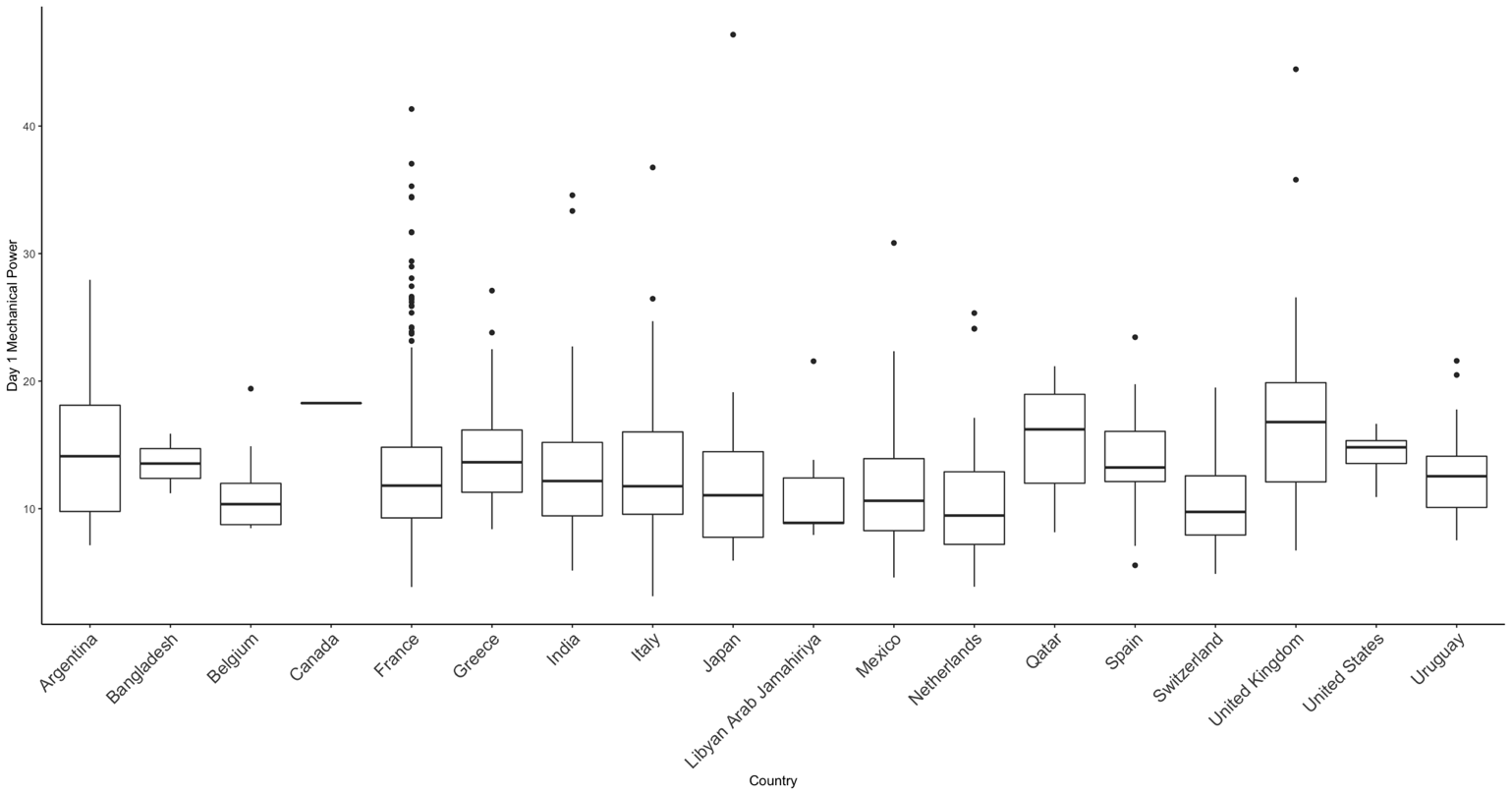


J/min

**Table S1: Demographics, comorbidities, baseline and clinical characteristics by hospital day**

|  |  | **HD1** | **HD3** | **HD7** |
| --- | --- | --- | --- | --- |
|  |  | **(N=1217)^#^** | **(N=866)^#^** | **(N=520)^#^** |
| **Baseline Characteristics** | |  |  |  |
| Age (years)^†^ |  | 51.2 (18.1) | 49.7 (18.1) | 50.7 (17.9) |
| Female |  | 412 (34 %) | 290 (33%) | 190 (37 %) |
| Height (cm)† | | 170 (9.17) | 170 (9.18) | 170 (9.22) |
| Weight (kg)† |  | 76.0 (16.2) | 76.4 (16.3) | 77.3 (16.9) |
| BMI (cm/kg)† | | 26.3 (5.14) | 26.3 (5.18) | 26.4 (5.19) |
| **Geographic region** | |  |  |  |
| Europe and Central Asia | | 845 (69 %) | 565 (65 %) | 367 (71 %) |
| Latin America & Caribbean | | 236 (19 %) | 200 (23 %) | 100 (19 %) |
| South Asia | | 75 (6 %) | 59 (7 %) | 32 (6 %) |
| Middle East and North Africa | | 25 (2 %) | 16 (2 %) | 10 (2 %) |
| East Asia and Pacific | | 29 (2 %) | 21 (2 %) | 9 (2 %) |
| North America | | 7 (1 %) | 5 (1 %) | 2 (0 %) |
| **Country Income Level**^†††^ | |  |  |  |
| High |  | 928 (76 %) | 625 (72 %) | 399 (77 %) |
| Upper middle | | 198 (19 %) | 214 (18 %) | 182 (21 %) |
| Lower  middle |  | 75 (6 %) | 59 (7 %) | 32 (6 %) |
| **Comorbidities** | |  |  |  |
| Hypertension | | 346 (28 %) | 224 (26 %) | 145 (28 %) |
| Diabetes |  | 104 (12 %) | 63 (12 %) | 146 (12 %) |
| Heart failure |  | 21 (2 %) | 15 (3 %) | 35 (3 %) |
| Pulmonary disease | | 48 (4 %) | 35 (4 %) | 25 (5 %) |
| Malignancy |  | 35 (4 %) | 15 (3 %) | 53 (4 %) |
| Current Tobacco Use | | 280 (23 %) | 204 (24 %) | 140 (27 %) |
| **Clinical characteristics** | |  |  |  |
| Type of Brain Injury | |  |  |  |
| Traumatic brain injury | | 588 (48 %) | 443 (51 %) | 261 (50 %) |
| Subarachnoid hemorrhage | | 218 (18 %) | 148 (17 %) | 103 (20 %) |
| Intracranial hemorrhage | | 382 (31 %) | 273 (32 %) | 173 (33 %) |
| Acute ischemic stroke | | 97 (8 %) | 57 (7 %) | 32 (6 %) |
| CNS infection | | 60 (5 %) | 48 (6 %) | 31 (6 %) |
| Brain tumor | | 60 (5 %) | 40 (5 %) | 13 (3 %) |
| Initial GCS^††^ |  | 7.00 [3.00, 12.0] | 7.00 [3.00, 12.0] | 7.00 [3.00, 12.0] |
| GCS eyes^††^ |  | 1.00 [1.00, 4.00] | 1.00 [1.00, 4.00] | 1.00 [1.00, 4.00] |
| GCS verbal^††^ |  | 1.00 [1.00, 4.00] | 1.00 [1.00, 5.00] | 1.00 [1.00, 5.00] |
| GCS motor^††^ |  | 4.00 [1.00, 6.00] | 4.00 [1.00, 6.00] | 4.00 [1.00, 6.00] |
| Anisocoria |  | 338 (28 %) | 253 (29 %) | 163 (31 %) |
| Posterior fossa injury | | 57 (6 %) | 70 (6 %) | 47 (5 %) |
| Nosocomial VAP | | 406 (39 %) | 480 (40 %) | 387 (45 %) |
| **ARDS** |  | **114 (9%)** | **102 (12 %)** | **86 (17%)** |
| Mild |  | 24 (21%) | 20 (20%) | 12 (13%) |
| Moderate |  | 43 (38%) | 39 (38%) | 37 (43%) |
| Severe |  | 47 (41%) | 42 (41%) | 38 (44%) |
| **Treatment modalities utilized** | |  |  |  |
| Intraparenchymal ICP monitor | | 504 (41 %) | 381 (44 %) | 261 (50 %) |
| Extraventricular drain | | 360 (30 %) | 258 (30 %) | 179 (34 %) |
| Decompressive craniectomy | | 218 (18 %) | 174 (20 %) | 108 (21 %) |
| Intracranial neurosurgery | | 483 (40 %) | 340 (39 %) | 199 (38 %) |
| Barbiturate coma | | 74 (6 %) | 63 (7 %) | 44 (8 %) |
| Therapeutic hypothermia | | 56 (5 %) | 53 (6 %) | 43 (8 %) |

Clinical characteristics based on hospital mortality.
# number of patients included in the multivariable analyses (for any outcome)

ARDS=acute respiratory distress syndrome, BMI=body mass index, CNS=central nervous system, GCS=Glasgow Coma Scale, ICP=intracranial pressure, VAP=ventilator associated pneumonia

^†^Mean (standard deviation), ^††^ Median (interquartile range), ^†††^ The Country Income Level was based on the World Health Organization.

**Tables S2: Mechanical power stratified by country**

**Hospital Day 1**

| **Country** | **Median (J/min)** | **IQR (J/min)** | **Range, min-max (J/min)** |
| --- | --- | --- | --- |
| Argentina | 14.11 | 9.78-18.11 | 7.13-27.94 |
| Bangladesh | 13.54 | 12.38-14.71 | 11.21-15.88 |
| Belgium | 10.36 | 8.75-11.98 | 8.46-19.40 |
| Canada | 18.28 | 18.28 | 18.28 |
| France | 11.80 | 9.27-14.81 | 3.85-41.33 |
| Greece | 13.64 | 11.29-16.17 | 8.39-27.09 |
| India | 12.17 | 9.44-15.20 | 5.15-34.57 |
| Italy | 11.76 | 9.56-16.02 | 3.14-36.75 |
| Japan | 11.05 | 7.76-14.47 | 5.93-47.16 |
| Libyan Arab Jamahiriya | 8.89 | 8.86-12.41 | 7.94-21.56 |
| Mexico | 10.62 | 8.28-13.93 | 4.61-30.83 |
| Netherlands | 9.46 | 7.21-12.89 | 3.88-25.34 |
| Qatar | 16.22 | 11.96-18.96 | 8.15-21.17 |
| Spain | 13.23 | 12.13-16.06 | 5.56-23.44 |
| Switzerland | 9.75 | 7.94-12.58 | 4.89-19.49 |
| United Kingdom | 16.79 | 6.73-19.87 | 6.73-44.45 |
| United States | 14.81 | 13.54-15.34 | 10.91-16.66 |
| Uruguay | 12.54 | 10.09-14.11 | 7.52-21.59 |

**Hospital Day 3**

| **Country** | **Median (J/min)** | **IQR (J/min)** | **Range, min-max (J/min)** | **Data not available (N)** |
| --- | --- | --- | --- | --- |
| Argentina | 14.07 | 9.88-18.63 | 6.56-26.46 | - |
| Bangladesh | NA | NA | NA | 2 |
| Belgium | 11.90 | 9.03-14.14 | 7.06-19.60 | 7 |
| Canada | NA | NA | NA | 1 |
| France | 12.70 | 10.01-17.59 | 3.23-39.16 | 205 |
| Greece | 12.74 | 11.03-17.42 | 7.52-32.34 | 3 |
| India | 12.59 | 9.45-16.48 | 5.89-33.92 | 14 |
| Italy | 12.99 | 10.29-15.55 | 5.93-31.17 | 19 |
| Japan | 11.06 | 8.61-15.29 | 6.11-37.00 | 8 |
| Libyan Arab Jamahiriya | 8.89 | 7.87-11.01 | 5.48-15.13 | - |
| Mexico | 11.87 | 9.46-15.09 | 2.64-27.44 | 32 |
| Netherlands | 7.21 | 5.42-11.75 | 2.57-24.59 | 15 |
| Qatar | 17.64 | 16.93-17.64 | 16.23-21.17 | 9 |
| Spain | 14.89 | 11.86-16.73 | 7.19-27.52 | 5 |
| Switzerland | 8.78 | 7.17-10.91 | 4.85-19.51 | 17 |
| United Kingdom | 15.27 | 11.14-21.79 | 7.68-33.63 | 9 |
| United States | 14.96 | 11.85-15.52 | 8.87-15.68 | 1 |
| Uruguay | 12.70 | 11.43-17.14 | 8.56-26.91 | 4 |

**Hospital Day 7**

| **Country** | **Median (J/min)** | **IQR (J/min)** | **Range, min-max (J/min)** | **Data not available (N)** |
| --- | --- | --- | --- | --- |
| Argentina | 17.45 | 14.37-19.48 | 8.47-34.67 | 27 |
| Bangladesh | NA | NA | NA | 2 |
| Belgium | 15.99 | 12.58-16.38 | 9.16-16.76 | 11 |
| Canada | NA | NA | NA | 1 |
| France | 15.72 | 11.41-21.48 | 3.56-66.99 | 338 |
| Greece | 13.95 | 9.62-19.04 | 5.61-25.87 | 7 |
| India | 13.53 | 8.94-17.51 | 5.83-34-34 | 41 |
| Italy | 13.45 | 10.70-20.74 | 5.41-33.20 | 30 |
| Japan | 13.59 | 11.43-15.49 | 6.11-21.61 | 20 |
| Libyan Arab Jamahiriya | 8.36 | 7.41-10.37 | 4.52-15.56 | 1 |
| Mexico | 12.79 | 9.05-16.46 | 5.21-37.59 | 97 |
| Netherlands | 8.66 | 6.23-11.11 | 3.57-34.99 | 19 |
| Qatar | NA | NA | NA | 14 |
| Spain | 15.53 | 14.34-19.69 | 8.39-32.39 | 12 |
| Switzerland | 12.37 | 10.80-16.94 | 5.55-29.86 | 33 |
| United Kingdom | 16.41 | 12.23-25.69 | 7.49-30.53 | 28 |
| United States | 19.18 | 15.19-23.17 | 11.20-27.17 | 4 |
| Uruguay | 14.41 | 12.49-17.99 | 9.78-28.98 | 12 |

**Table S3a:** Multivariable analysis, Hospital mortality

**Mechanical power, Hospital day 1**

Number of obs = 1,113

Deviance = 399.9057473 (1/df) Deviance = .3609258

Pearson = 861.8162742 (1/df) Pearson = .7778125

(Std. Err. adjusted for 5 clusters in region)

| **Hospital Mortality** | Coefficient | Robust Std. Err | z | P> [z] | 95% Confidence Interval |
| --- | --- | --- | --- | --- | --- |
| Mechanical Power, HD 1 | .0301043 | .0052722 | 5.71 | 0.000 | .019771 .0404377 |
| Age | .0264753 | .0115868 | 2.28 | 0.022 | .0037656 .0491849 |
| Body Mass Index | -.0453969 | .0093164 | -4.87 | 0.000 | -.0636567 -.0271372 |
| H/o Heart Failure | .5191577 | .090322 | 5.75 | 0.000 | .3421298 .6961856 |
| H/o Diabetes | .3811028 | .0790038 | 4.82 | 0.000 | .2262582 .5359474 |
| H/o Pulmonary Disease | -1.192107 | .12937 | -9.21 | 0.000 | -1.445668 -.9385466 |
| Initial GCS | -.0706031 | .0188831 | -3.74 | 0.000 | -.1076132 -.0335929 |
| Traumatic Brain Injury | -.113926 | .2195373 | -0.52 | 0.604 | -.5442113 .3163592 |
| Subarachnoid Hemorrhage | .1834075 | .0884602 | 2.07 | 0.038 | .0100288 .3567863 |
| CNS Infection | .602636 | .2883635 | 2.09 | 0.037 | .0374538 1.167818 |
| Anisocoria | .196991 | .0924642 | 2.13 | 0.033 | .0157645 .3782175 |
| Barbiturate Coma | -.4426161 | .5062048 | -0.87 | 0.382 | -1.434759 .5495272 |
| Intracranial ICP Monitor | -.6323134 | .0367143 | -17.22 | 0.000 | -.7042721 -.5603548 |
| External Ventricular Drain | .343978 | .0668197 | 5.15 | 0.000 | .2130138 .4749421 |
| Decompressive Craniectomy | .5637832 | .103267 | 5.46 | 0.000 | .3613836 .7661828 |
| Propofol HD 1 | -.4598246 | .4102576 | -1.12 | 0.262 | -1.263915 .3442656 |
| Midazolam HD 1 | -.4839317 | .3101394 | -1.56 | 0.119 | -1.091794 .1239304 |
| HD1 Ventilator Mode | .3435152 | .1613061 | 2.13 | 0.033 | .027361 .6596694 |
| P/F ratio HD 1 | -828.9449 | 803.5588 | -1.03 | 0.302 | -2403.891 746.0015 |
| Constant | -1.918585 | .7812687 | -2.46 |  | -3.449844 -.3873269 |

GCS=Glasgow Coma Scale, HD=Hospital Day, h/o= prior medical history of, ICP=Intracranial Pressure, P/F = pressure of arterial oxygen/fraction of inspired oxygen

chi2( 1) = 32.60, Prob > chi2 = 0.0000

**Mechanical power, Hospital day 3**

No. of obs = 688

Deviance = 64.84594699 (1/df) Deviance = .094804

Pearson = 64.84594699 (1/df) Pearson = .094804

(Std. Err. adjusted for 5 clusters in region)

| **Hospital Mortality** | Coefficient | Robust Std. Err | z | P> [z] | 95% Confidence Interval |
| --- | --- | --- | --- | --- | --- |
| Mechanical Power, HD 3 | -.6507107 | .1545198 | -4.21 | 0.000 | -.953564 -.3478574 |
| Age | .0383603 | .023104 | 1.66 | 0.097 | -.0069227 .0836433 |
| Body Mass Index | -.0996223 | .0417042 | -2.39 | 0.017 | -.181361 -.0178835 |
| H/o Heart Failure | -.1134211 | .1587448 | -0.71 | 0.475 | -.4245552 .197713 |
| H/o Diabetes | .2338762 | .1057158 | 2.21 | 0.027 | .0266769 .4410754 |
| H/o Pulmonary Disease | -1.351204 | .8085927 | -1.67 | 0.095 | -2.936017 .2336082 |
| Initial GCS | -.0798217 | .0332364 | -2.40 | 0.016 | -.144964 -.0146795 |
| Traumatic Brain Injury | .3124267 | .3798759 | 0.82 | 0.411 | -.4321164 1.05697 |
| Subarachnoid Hemorrhage | .0607375 | .1748359 | 0.35 | 0.728 | -.2819346 .4034096 |
| CNS Infection | .5756343 | .1486883 | 3.87 | 0.000 | .2842106 .8670579 |
| Anisocoria | .3326659 | .2496401 | 1.33 | 0.183 | -.1566197 .8219514 |
| Barbiturate Coma | -.6846639 | .2239412 | -3.06 | 0.002 | -1.123581 -.2457471 |
| Intracranial ICP Monitor | -.781299 | .1527528 | -5.11 | 0.000 | -1.080689 -.481909 |
| External Ventricular Drain | .6112948 | .138558 | 4.41 | 0.000 | .3397261 .8828635 |
| Decompressive Craniectomy | .7614202 | .2414871 | 3.15 | 0.002 | .2881142 1.234726 |
| Propofol HD 3 | -.1055046 | .3793411 | -0.28 | 0.781 | -.8489994 .6379902 |
| Midazolam HD 3 | -.530344 | .3302494 | -1.61 | 0.108 | -1.177621 .1169329 |
| HD3 Ventilator Mode | .6309858 | .0648397 | 9.73 | 0.000 | .5039024 .7580692 |
| P/F ratio HD 3 | 1.70e-09 | 1.19e-09 | 1.43 | 0.154 | -6.37e-10 4.03e-09 |
| Constant | -1.622139 | 1.151058 | -1.41 | 0.159 | -3.878171 .633893 |

GCS=Glasgow Coma Scale, HD=Hospital Day, h/o= prior medical history of, ICP=Intracranial Pressure, P/F = pressure of arterial oxygen/fraction of inspired oxygen

chi2( 1) = 17.73, Prob > chi2 = 0.0000

**Mechanical power, Hospital day 7**

No. of obs = 391

Deviance = 149.4886394 (1/df) Deviance = .3862756

Pearson = 269.5797555 (1/df) Pearson = .6965885

(Std. Err. adjusted for 5 clusters in region)

| **Hospital Mortality** | Coefficient | Robust Std. Err | z | P> [z] | 95% Confidence Interval |
| --- | --- | --- | --- | --- | --- |
| Mechanical Power, HD 7 | -22.33042 | 7.696738 | -2.90 | 0.004 | -37.41575 -7.24509 |
| Age | .0257592 | .0104948 | 2.45 | 0.014 | .0051898 .0463286 |
| Body Mass Index | -.0629116 | .0261696 | -2.40 | 0.016 | -.114203 -.0116201 |
| H/o Heart Failure | .0383919 | .2525395 | 0.15 | 0.879 | -.4565764 .5333602 |
| H/o Diabetes | .5123256 | .1519701 | 3.37 | 0.001 | .2144698 .8101815 |
| H/o Pulmonary Disease | -.3160703 | .1331239 | -2.37 | 0.018 | -.5769883 -.0551523 |
| Initial GCS | -.0440152 | .0233137 | -1.89 | 0.059 | -.0897093 .0016789 |
| Traumatic Brain Injury | .330342 | .3399538 | 0.97 | 0.331 | -.3359552 .9966392 |
| Subarachnoid Hemorrhage | .2316865 | .2718527 | 0.85 | 0.394 | -.3011349 .7645079 |
| CNS Infection | .7745609 | .3676655 | 2.11 | 0.035 | .0539498 1.495172 |
| Anisocoria | .2218538 | .1118429 | 1.98 | 0.047 | .0026457 .441062 |
| Barbiturate Coma | -1.425385 | .168155 | -8.48 | 0.000 | -1.754963 -1.095807 |
| Intracranial ICP Monitor | -.7091122 | .1243405 | -5.70 | 0.000 | -.9528151 -.4654093 |
| External Ventricular Drain | .6468752 | .1321119 | 4.90 | 0.000 | .3879405 .9058098 |
| Decompressive Craniectomy | .5351293 | .1693278 | 3.16 | 0.002 | .203253 .8670057 |
| Propofol HD 7 | -.5630445 | .2584827 | -2.18 | 0.029 | -1.069661 -.0564278 |
| Midazolam HD 7 | -.2340482 | .2661886 | -0.88 | 0.379 | -.7557683 .2876719 |
| HD7 Ventilator Mode | .114616 | .2957703 | 0.39 | 0.698 | -.4650832 .6943151 |
| P/F ratio HD7 | -.0529184 | .0290269 | -1.82 | 0.068 | -.1098101 .0039733 |
| Constant | -.6378144 | .7881972 | -0.81 | 0.418 | -2.182653 .9070237 |

GCS=Glasgow Coma Scale, HD=Hospital Day, h/o= prior medical history of, ICP=Intracranial Pressure, P/F = pressure of arterial oxygen/fraction of inspired oxygen

chi2( 1) = 8.42, Prob > chi2 = 0.0037

**Table S3b:** Multivariable analysis, Need for Reintubation

**Mechanical power, Hospital day 1**

No. of obs = 803

Deviance = 392.8079678 (1/df) Deviance = .4922406

Pearson = 532.717375 (1/df) Pearson = .6675656

(Std. Err. adjusted for 5 clusters in region)

| **Reintubation** | Coefficient | Robust Std. Err | z | P> [z] | 95% Confidence Interval |
| --- | --- | --- | --- | --- | --- |
| Mechanical Power, HD 1 | .0993263 | .0083133 | 11.95 | 0.000 | .0830326 .1156201 |
| Age | .0066804 | .0024372 | 2.74 | 0.006 | 0019035 .0114572 |
| Body Mass Index | -.0246939 | .0072458 | -3.41 | 0.001 | -.0388954 -.0104924 |
| H/o Heart Failure | -1.039331 | .4272758 | -2.43 | 0.015 | -1.876776 -.201886 |
| H/o Hypertension | -.0795448 | .0553665 | -1.44 | 0.151 | -.188061 .0289715 |
| H/o Pulmonary Disease | .4780761 | .084964 | 5.63 | 0.000 | .3115497 .6446026 |
| Initial GCS | -.0741891 | .0099953 | -7.42 | 0.000 | -.0937795 -.0545988 |
| Traumatic Brain Injury | -.2487515 | .0758829 | -3.28 | 0.001 | -.3974793 -.1000237 |
| Intracranial Hemorrhage | .2206979 | .0530091 | 4.16 | 0.000 | .1168019 .3245938 |
| Anisocoria | -.0330333 | .058506 | -0.56 | 0.572 | -.1477029 .0816364 |
| Barbiturate Coma | -.2190308 | .3017589 | -0.73 | 0.468 | -.8104675 .3724059 |
| Intracranial ICP Monitor | -.212089 | .056735 | -3.74 | 0.000 | -.3232875 -.1008905 |
| External Ventricular Drain | .2107422 | .0859024 | 2.45 | 0.014 | .0423767 .3791078 |
| Propofol HD 1 | -.0319441 | .1579392 | -0.20 | 0.840 | -.3414992 .2776111 |
| Midazolam HD 1 | .1137782 | .0937495 | 1.21 | 0.225 | -.0699674 .2975239 |
| HD1 Ventilator Mode | .0494568 | .0327885 | 1.51 | 0.131 | -.0148075 .1137212 |
| P/F ratio HD 1 1 | -12185 | 5016.153 | -2.43 | 0.015 | -22016.47 -2353.517 |
| P/F ratio HD 1 2 | 206.3016 | 78.75681 | 2.62 | 0.009 | 51.94111 360.6621 |
| Swallowing Attempts | -.4649306 | .2226972 | -2.09 | 0.037 | -.9014091 -.0284522 |
| Frequency of ETT suctioning | .1808568 | .0831185 | 2.18 | 0.030 | .0179475 .343766 |
| Constant | -1.039157 | .4085676 | -2.54 | 0.011 | -1.839935 -.2383797 |

ETT= Endotracheal Tube, GCS=Glasgow Coma Scale, HD=Hospital Day, h/o= prior medical history of, ICP=Intracranial Pressure, P/F = pressure of arterial oxygen/fraction of inspired oxygen

chi2( 1) = 142.75, Prob > chi2 = 0.0000

**Mechanical power, Hospital day 3**

No. of obs = 460

Deviance = 208.1301202 (1/df) Deviance = .4574288

Pearson = 300.4082309 (1/df) Pearson = .6602379

(Std. Err. adjusted for 5 clusters in region)

| **Reintubation** | Coefficient | Robust Std. Err | z | P> [z] | 95% Confidence Interval |
| --- | --- | --- | --- | --- | --- |
| Mechanical Power, HD 3 | .0159331 | .0031956 | 4.99 | 0.000 | .0096698 .0221963 |
| Age | .0168325 | .002105 | 8.00 | 0.000 | .0127069 .0209582 |
| Body Mass Index | .0226369 | .0054601 | 4.15 | 0.000 | .0119353 .0333386 |
| H/o Heart Failure | -7.170192 | .4815191 | -14.89 | 0.000 | -8.113952 -6.226432 |
| H/o Hypertension | -.1356586 | .1319576 | -1.03 | 0.304 | -.3942908 .1229737 |
| H/o Pulmonary Disease | .1552892 | .2381886 | 0.65 | 0.514 | -.3115519 .6221303 |
| Initial GCS | -.0180725 | .005769 | -3.13 | 0.002 | -.0293795 -.0067654 |
| Traumatic Brain Injury | -.1910051 | .0488833 | -3.91 | 0.000 | -.2868146 -.0951956 |
| Intracranial Hemorrhage | .3150729 | .1171877 | 2.69 | 0.007 | .0853893 .5447565 |
| Anisocoria | .0105653 | .0863387 | 0.12 | 0.903 | -.1586554 .1797861 |
| Barbiturate Coma | .0583885 | .7275371 | 0.08 | 0.936 | -1.367558 1.484335 |
| Intracranial ICP Monitor | -.6903188 | .3088228 | -2.24 | 0.025 | -1.2956 -.0850372 |
| External Ventricular Drain | .085109 | .1667603 | 0.51 | 0.610 | -.2417353 .4119533 |
| Propofol HD 3 | .3260763 | .1540158 | 2.12 | 0.034 | .0242108 .6279418 |
| Midazolam HD 3 | .1827412 | .2981706 | 0.61 | 0.540 | -.4016624 .7671448 |
| HD3 Ventilator Mode | -.1579792 | .3220312 | -0.49 | 0.624 | -.7891488 .4731904 |
| P/F ratio HD 3 1 | -18033.36 | 2403.273 | -7.50 | 0.000 | -22743.68 -13323.03 |
| P/F ratio HD 3 2 | 5435.966 | 725.5819 | 7.49 | 0.000 | 4013.851 6858.08 |
| Swallowing Attempts | -.8531576 | .3297103 | -2.59 | 0.010 | -1.499378 -.2069372 |
| Frequency of ETT suctioning | .3901587 | .1129455 | 3.45 | 0.001 | .1687895 .6115278 |
| Cons | -2.543815 | .4166433 | -6.11 | 0.000 | -3.360421 -1.727209 |

ETT= Endotracheal Tube, GCS=Glasgow Coma Scale, HD=Hospital Day, h/o= prior medical history of, ICP=Intracranial Pressure, P/F = pressure of arterial oxygen/fraction of inspired oxygen

chi2( 1) = 24.86, Prob > chi2 = 0.0000

**Mechanical power, Hospital day 7**

No. of obs = 208

Deviance = 95.24333818 (1/df) Deviance = .4646016

Pearson = 115.5081308 (1/df) Pearson = .5634543

(Std. Err. adjusted for 4 clusters in region)

| **Reintubation** | Coefficient | Robust Std. Err | z | P> [z] | 95% Confidence Interval |
| --- | --- | --- | --- | --- | --- |
| Mechanical Power, HD 7 | -68.30709 | 17.55865 | -3.89 | 0.000 | 102.7214 -33.89276 |
| Age | .0045169 | .0019712 | 2.29 | 0.022 | .0006534 .0083805 |
| Body Mass Index | -.0516073 | 0199591 | -2.59 | 0.010 | -.0907263 -.0124882 |
| H/o Heart Failure | .9840627 | .0942126 | 10.45 | 0.000 | .7994095 1.168716 |
| H/o Hypertension | -.4523078 | .2105705 | -2.15 | 0.032 | -.8650184 -.0395972 |
| H/o Pulmonary Disease | .3789682 | .1610949 | 2.35 | 0.019 | .063228 .6947085 |
| Initial GCS | -.0037186 | .0368867 | -0.10 | 0.920 | -.0760151 .0685779 |
| Traumatic Brain Injury | -.7816418 | .5097659 | -1.53 | 0.125 | -1.780765 .2174811 |
| Intracranial Hemorrhage | .2465613 | .0723005 | 3.41 | 0.001 | .1048549 .3882677 |
| Anisocoria | .1751034 | .3210091 | 0.55 | 0.585 | -.4540629 .8042698 |
| Barbiturate Coma | -.6485742 | 2.150263 | -0.30 | 0.763 | -4.863012 3.565864 |
| Intracranial ICP Monitor | .0539009 | .283901 | 0.19 | 0.849 | -.5025348 .6103366 |
| External Ventricular Drain | -.4882817 | .2735857 | -1.78 | 0.074 | -1.0245 .0479364 |
| Propofol HD 7 | -.4627596 | .1918562 | -2.41 | 0.016 | -.838791 -.0867283 |
| Midazolam HD 7 | .0025163 | .0918179 | 0.03 | 0.978 | -.1774435 .1824761 |
| HD7 Ventilator Mode | .1577739 | .4512202 | 0.35 | 0.727 | -.7266015 1.042149 |
| P/F ratio HD 7 1 | -68.30709 | 17.55865 | -3.89 | 0.000 | -102.7214 -33.89276 |
| P/F ratio HD 7 2 | 645.5641 | 315.5699 | 2.05 | 0.041 | 27.05844 1264.07 |
| Swallowing Attempts | -.0940412 | .4852527 | -0.19 | 0.846 | -1.045119 .8570365 |
| Frequency of ETT suctioning | .4326083 | .1823795 | 2.37 | 0.018 | .0751511 .7900654 |
| Constant | .3870961 | .8453571 | 0.46 | 0.647 | -1.269773 2.043966 |

ETT= Endotracheal Tube, GCS=Glasgow Coma Scale, HD=Hospital Day, h/o= prior medical history of, ICP=Intracranial Pressure, P/F = pressure of arterial oxygen/fraction of inspired oxygen

chi2( 1) = 15.13, Prob > chi2 = 0.0001

**Table S3c:** Multivariable analysis, Tracheostomy Placement

**Mechanical power, Hospital day 1**

No. of obs = 803

Deviance = 244.3664725 (1/df) Deviance = .3058404

Pearson = 688.8596036 (1/df) Pearson = .8621522

(Std. Err. adjusted for 5 clusters in region)

| **Tracheostomy placement** | Coefficient | Robust Std. Err | z | P> [z] | 95% Confidence Interval |
| --- | --- | --- | --- | --- | --- |
| Mechanical Power, HD 1 | -.2037581 | .0720168 | -2.83 | 0.005 | -.3449084 -.0626077 |
| Age | .0049927 | .002966 | 1.68 | 0.092 | -.0008205 .0108059 |
| Body Mass Index | -.0047686 | .0152042 | -0.31 | 0.754 | -.0345683 .0250311 |
| H/o Heart Failure | -1.043695 | .4979417 | -2.10 | 0.036 | -2.019643 -.0677472 |
| H/o Hypertension | -.2575515 | .1769905 | -1.46 | 0.146 | -.6044466 .0893436 |
| H/o Pulmonary Disease | .3840189 | .1272019 | 3.02 | 0.003 | .1347077 .6333301 |
| Initial GCS | -.0941744 | .0399625 | -2.36 | 0.018 | -.1724995 -.0158494 |
| Traumatic Brain Injury | .1510669 | .0984437 | 1.53 | 0.125 | -.0418792 .344013 |
| Intracranial Hemorrhage | .0698163 | .1361204 | 0.51 | 0.608 | -.1969748 .3366074 |
| Anisocoria | -.0719011 | .1593936 | -0.45 | 0.652 | -.3843068 .2405046 |
| Barbiturate Coma | -1.039708 | .2448754 | -4.25 | 0.000 | -1.519655 -.5597609 |
| Intracranial ICP Monitor | -.6772633 | .145399 | -4.66 | 0.000 | -.96224 -.3922865 |
| External Ventricular Drain | .3984889 | .0472049 | 8.44 | 0.000 | .305969 .4910089 |
| Propofol HD 1 | -.1172618 | .1609405 | -0.73 | 0.466 | -.4326993 .1981758 |
| Midazolam HD 1 | .2837204 | .1737388 | 1.63 | 0.102 | -.0568014 .6242422 |
| HD1 Ventilator Mode | -.2937243 | .1984946 | -1.48 | 0.139 | -.6827665 .095318 |
| P/F ratio HD 1_1 | -70670.71 | 23155.92 | -3.05 | 0.002 | -116055.5 -25285.95 |
| P/F ratio HD 1_2 | 18001.13 | 5499.731 | 3.27 | 0.001 | 7221.856 28780.4 |
| Swallowing Attempts | -.3144642 | .5544719 | -0.57 | 0.571 | -1.401209 .7722808 |
| Frequency of ETT suctioning | .1312937 | .1883759 | 0.70 | 0.486 | -.2379163 .5005037 |
| Constant | -1.966788 | .3952294 | -4.98 | 0.000 | -2.741424 -1.192153 |

ETT= Endotracheal Tube, GCS=Glasgow Coma Scale, HD=Hospital Day, h/o= prior medical history of, ICP=Intracranial Pressure, P/F = pressure of arterial oxygen/fraction of inspired oxygen

**Mechanical power, Hospital day 3**

No. of obs = 461

Deviance = 132.7866258 (1/df) Deviance = .2911987

Pearson = 339.3604635 (1/df) Pearson = .7442115

(Std. Err. adjusted for 5 clusters in region)

| **Tracheostomy placement** | Coefficient | Robust Std. Err | z | P> [z] | 95% Confidence Interval |
| --- | --- | --- | --- | --- | --- |
| Mechanical Power, HD 3 | .0245883 | .0105093 | 2.34 | 0.019 | .0039904 .0451862 |
| Age | .0196478 | .0040919 | 4.80 | 0.000 | .0116278 .0276678 |
| Body Mass Index | .0193729 | .0119939 | 1.62 | 0.106 | -.0041347 .0428804 |
| H/o Heart Failure | -15.04775 | 1.00722 | -14.94 | 0.000 | -17.02186 -13.07363 |
| H/o Hypertension | -.8545545 | .4970047 | -1.72 | 0.086 | -1.828666 .1195569 |
| H/o Pulmonary Disease | .3809167 | .3792668 | 1.00 | 0.315 | -.3624325 1.124266 |
| Initial GCS | -.0643829 | .018865 | -3.41 | 0.001 | -.1013576 -.0274082 |
| Traumatic Brain Injury | .2440691 | .1407863 | 1.73 | 0.083 | -.031867 .5200052 |
| Intracranial Hemorrhage | .3117952 | .0359308 | 8.68 | 0.000 | .2413721 .3822183 |
| Anisocoria | .22991 | .0827418 | 2.78 | 0.005 | .067739 .3920809 |
| Barbiturate Coma | -.8468753 | .156953 | -5.40 | 0.000 | -1.154498 -.539253 |
| Intracranial ICP Monitor | -1.568642 | .2502191 | -6.27 | 0.000 | 2.059062 -1.078221 |
| External Ventricular Drain | -.0521705 | .1146173 | -0.46 | 0.649 | -.2768162 .1724752 |
| Propofol HD 3 | .0496081 | .1355855 | 0.37 | 0.714 | -.2161345 .3153507 |
| Midazolam HD 3 | .4076388 | .2867888 | 1.42 | 0.155 | -.1544569 .9697344 |
| HD3 Ventilator Mode | -.1125617 | .3262059 | -0.35 | 0.730 | -.7519135 .52679 |
| P/F ratio HD 3 1 | -30414.15 | 6259.467 | -4.86 | 0.000 | -42682.48 -18145.82 |
| P/F ratio HD 3 2 | 8968.632 | 1897.263 | 4.73 | 0.000 | 5250.065 12687.2 |
| Swallowing Attempts | -.9330956 | .3514229 | -2.66 | 0.008 | -1.621872 -.2443194 |
| Frequency of ETT suctioning | .3828844 | .1986584 | 1.93 | 0.054 | -.0064789 .7722478 |
| Constant | -2.981673 | .3293234 | -9.05 | 0.000 | -3.627135 -2.336211 |

ETT= Endotracheal Tube, GCS=Glasgow Coma Scale, HD=Hospital Day, h/o= prior medical history of, ICP=Intracranial Pressure, P/F = pressure of arterial oxygen/fraction of inspired oxygen

chi2( 1) = 5.47, Prob > chi2 = 0.0193

**Mechanical power, Hospital day 7**

Deviance = 78.58456764 (1/df) Deviance = .3572026

Pearson = 152.554057 (1/df) Pearson = .6934275

(Std. Err. adjusted for 4 clusters in region)

| **Tracheostomy placement** | Coefficient | Robust Std. Err | z | P> [z] | 95% Confidence Interval |
| --- | --- | --- | --- | --- | --- |
| Mechanical Power, HD 7 | -57.78876 | 18.12274 | 3.19 | 0.001 | -93.30868 -22.26884 |
| Age | .0100182 | .0022056 | 4.54 | 0.000 | .0056954 .014341 |
| Body Mass Index | .0142764 | .0070299 | 2.03 | 0.042 | .000498 .0280548 |
| H/o Heart Failure | -11.94846 | 1.20726 | -9.90 | 0.000 | -14.31465 -9.582274 |
| H/o Hypertension | -.6774109 | .1512272 | -4.48 | 0.000 | -.9738106 -.3810111 |
| H/o Pulmonary Disease | -.2519977 | .1392942 | -1.81 | 0.070 | -.5250094 .021014 |
| Initial GCS | -.0428087 | .0109356 | -3.91 | 0.000 | -.064242 -.0213754 |
| Traumatic Brain Injury | -.2494192 | .7533785 | -0.33 | 0.741 | -1.726014 1.227175 |
| Intracranial Hemorrhage | -.2401547 | .4251929 | -0.56 | 0.572 | -1.073517 .593208 |
| Anisocoria | .1169326 | .0707286 | 1.65 | 0.098 | -.0216929 .2555581 |
| Barbiturate Coma | -.9233831 | .2306855 | -4.00 | 0.000 | -1.375518 -.4712479 |
| Intracranial ICP Monitor | -1.308038 | .3536049 | -3.70 | 0.000 | -2.001091 -.614985 |
| External Ventricular Drain | -.2333406 | .6313783 | -0.37 | 0.712 | -1.470819 1.004138 |
| Propofol HD 7 | -.3479289 | .0549196 | -6.34 | 0.000 | -.4555694 -.2402885 |
| Midazolam HD 7 | .9771839 | .5180871 | 1.89 | 0.059 | -.0382481 1.992616 |
| HD7 Ventilator Mode | -1.198722 | .3013539 | -3.98 | 0.000 | -1.789365 -.6080797 |
| P/F ratio HD 7 | -2.12e-06 | 3.64e-06 | -0.58 | 0.560 | -9.26e-06 5.01e-06 |
| Swallowing Attempts | -.6198354 | .2881548 | -2.15 | 0.031 | -1.184608 -.0550624 |
| Frequency of ETT suctioning | .0703821 | .1467207 | 0.48 | 0.631 | -.2171852 .3579493 |
| Constant | -.546602 | .304762 | -1.79 | 0.073 | -1.143925 .0507206 |

ETT= Endotracheal Tube, GCS=Glasgow Coma Scale, HD=Hospital Day, h/o= prior medical history of, ICP=Intracranial Pressure, P/F = pressure of arterial oxygen/fraction of inspired oxygen

chi2( 1) = 10.17, Prob > chi2 = 0.0014

**Table S3d:** Multivariable analysis, Moderate to Severe Acute Respiratory Distress Syndrome (ARDS)

**Mechanical power, Hospital day 1**

No. of obs = 1,165

Deviance = 275.8447883 (1/df) Deviance = .2377972

Pearson = 844.9085848 (1/df) Pearson = .7283695

(Std. Err. adjusted for 6 clusters in region)

| **ARDS** | Coefficient | Robust Std. Err | z | P> [z] | 95% Confidence Interval |
| --- | --- | --- | --- | --- | --- |
| Mechanical Power HD 1 | 1.000734 | .1998367 | 5.01 | 0.000 | .6090614 1.392407 |
| Age | -.0080944 | .0042546 | -1.90 | 0.057 | -.0164333 .0002445 |
| Sex | -.3047529 | .2145255 | -1.42 | 0.155 | -.7252152 .1157094 |
| H/o Heart Failure | .5843033 | .6162296 | 0.95 | 0.343 | -.6234845 1.792091 |
| H/o Pulmonary Disease | .3569982 | .2436566 | 1.47 | 0.143 | -.1205599 .8345563 |
| Initial GCS | .0240601 | .023622 | 1.02 | 0.308 | -.0222382 .0703585 |
| Traumatic Brain Injury | .1764689 | .1605303 | 1.10 | 0.272 | -.1381646 .4911025 |
| Intracranial Hemorrhage | -.339384 | .0919008 | -3.69 | 0.000 | -.5195063 -.1592617 |
| Subarachnoid Hemorrhage | .6493831 | .1176772 | 5.52 | 0.000 | .41874 .8800262 |
| Decompressive Craniectomy | .494194 | .1240583 | 3.98 | 0.000 | 2510443 .7373437 |
| Nosocomial VAP | 1.995096 | .1535323 | 12.99 | 0.000 | 1.694178 2.296014 |
| HD1 PaO2 | -.6808599 | .1280838 | -5.32 | 0.000 | -.9318997 -.4298202 |
| HD1 PaCO2 | .0834002 | .0264993 | -3.15 | 0.002 | -.1353378 -.0314625 |
| HD1 Ventilator Mode | .9473856 | .1937134 | 4.89 | 0.000 | .5677144 1.327057 |
| Constant | -4.044748 | .1619326 | -24.98 | 0.000 | -4.36213 -3.727366 |

GCS=Glasgow Coma Scale, HD=Hospital Day, h/o= prior medical history of, PaO2 = pressure of arterial oxygen, PaCO2= pressure of arterial carbon dioxide

chi2( 1) = 25.08, Prob > chi2 = 0.0000

**Mechanical power, Hospital day 3**

No. of obs = 816

Deviance = 223.0202532 (1/df) Deviance = .2746555

Pearson = 619.6687875 (1/df) Pearson = .7631389

(Std. Err. adjusted for 5 clusters in region)

| **ARDS** | Coefficient | Robust Std. Err | z | P> [z] | 95% Confidence Interval |
| --- | --- | --- | --- | --- | --- |
| Mechanical Power HD 3 | .187923 | .0384116 | 4.89 | 0.000 | .1126376 .2632085 |
| Age | -.0016322 | .0040056 | -0.41 | 0.684 | -.009483 .0062186 |
| Sex | -.1731382 | .2420099 | -0.72 | 0.474 | -.647469 .3011926 |
| H/o Heart Failure | .544343 | .5637628 | 0.97 | 0.334 | -.5606119 1.649298 |
| H/o Pulmonary Disease | .8742049 | .2390524 | 3.66 | 0.000 | .4056707 1.342739 |
| Initial GCS | .0530401 | .0306112 | 1.73 | 0.083 | -.0069567 .1130368 |
| Traumatic Brain Injury | .2298116 | .1736494 | 1.32 | 0.186 | -.1105351 .5701582 |
| Intracranial Hemorrhage | -.3132573 | .1497822 | -2.09 | 0.036 | -.6068251 -.0196896 |
| Subarachnoid Hemorrhage | .7270907 | .1995322 | 3.64 | 0.000 | .3360147 1.118167 |
| Decompressive Craniectomy | .4264105 | .1325988 | 3.22 | 0.001 | .1665215 .6862995 |
| Nosocomial VAP | 1.606275 | .1242085 | 12.93 | 0.000 | 1.362831 1.849719 |
| HD3 PaO2 | .6765159 | .2844329 | 2.38 | 0.017 | .1190377 1.233994 |
| HD3 PaCO2 | -.0457863 | .0219938 | -2.08 | 0.037 | -.0888933 -.0026792 |
| HD3 Ventilator Mode | 1.032615 | .1521548 | 6.79 | 0.000 | .7343973 1.330833 |
| Constant | -5.421041 | .6374007 | -8.50 | 0.000 | -6.670323 -4.171758 |
